# Integration of Fungus-Specific CandA-C1 into a Trimeric CandA Complex Allowed Splitting of the Gene for the Conserved Receptor Exchange Factor of CullinA E3 Ubiquitin Ligases in Aspergilli

**DOI:** 10.1128/mBio.01094-19

**Published:** 2019-06-18

**Authors:** Anna M. Köhler, Rebekka Harting, Annika E. Langeneckert, Oliver Valerius, Jennifer Gerke, Cindy Meister, Anja Strohdiek, Gerhard H. Braus

**Affiliations:** a Department of Molecular Microbiology and Genetics, Institute of Microbiology and Genetics, Goettingen Center for Molecular Biosciences (GZMB), University of Göttingen, Göttingen, Germany; Universidad de Córdoba; Universidade de Sao Paulo; Charite/Humboldt University, Berlin and Xiamen University, China

**Keywords:** *Aspergillus fumigatus*, *Aspergillus nidulans*, COP9 signalosome, Cand1, Cullin-RING ubiquitin ligase, Nedd8, asexual development, protein complex, protein degradation, secondary metabolism, sexual development, spore germination

## Abstract

E3 cullin-RING ubiquitin ligase (CRL) complexes recognize specific substrates and are activated by covalent modification with ubiquitin-like Nedd8. Deneddylation inactivates CRLs and allows Cand1/A to bind and exchange substrate recognition subunits. Human as well as most fungi possess a single gene for the receptor exchange factor Cand1, which is split and rearranged in aspergilli into two genes for separate proteins. Aspergillus nidulans CandA-N blocks the neddylation site, and CandA-C inhibits the interaction to the adaptor/substrate receptor subunits similar to the respective N-terminal and C-terminal parts of single Cand1. The pathogen Aspergillus fumigatus and related species express a CandA-C with a 190-amino-acid N-terminal extension domain encoded by an additional exon. This extension corresponds in most aspergilli, including A. nidulans, to a gene directly upstream of *candA-C* encoding a 20-kDa protein without human counterpart. This protein was named CandA-C1, because it is also required for the cellular deneddylation/neddylation cycle and can form a trimeric nuclear complex with CandA-C and CandA-N. CandA-C and CandA-N are required for asexual and sexual development and control a distinct secondary metabolism. CandA-C1 and the corresponding domain of A. fumigatus control spore germination, vegetative growth, and the repression of additional secondary metabolites. This suggests that the dissection of the conserved Cand1-encoding gene within the genome of aspergilli was possible because it allowed the integration of a fungus-specific protein required for growth into the CandA complex in two different gene set versions, which might provide an advantage in evolution.

## Abstract (precis)

Aspergillus species are important for biotechnological applications, like the production of citric acid or antibacterial agents. Aspergilli can cause food contamination or invasive aspergillosis to immunocompromised humans or animals. Specific treatment is difficult due to limited drug targets and emerging resistances. The CandA complex regulates, as a receptor exchange factor, the activity and substrate variability of the ubiquitin labeling machinery for 26S proteasome-mediated protein degradation. Only *Aspergillus* species encode at least two proteins that form a CandA complex. This study shows that *Aspergillus* species had to integrate a third component into the CandA receptor exchange factor complex that is unique to aspergilli and required for vegetative growth, sexual reproduction, and activation of the ubiquitin labeling machinery. These features have interesting implications for the evolution of protein complexes and could make CandA-C1 an interesting candidate for target-specific drug design to control fungal growth without affecting the human ubiquitin-proteasome system.

## INTRODUCTION

Aspergilli are filamentous ascomycetes which can differentiate into an asexual and sexual spore-producing life cycle (1, 2). The 350 known Aspergillus species are ubiquitously distributed, mostly saprophytic in soil, and have harmful and beneficial properties to plants, animals, and humans (2). Some species are useful in biotechnology and the pharma industry through secondary metabolite production, like cholesterol-reducing lovastatin from Aspergillus terreus (3). Aspergillus nidulans is used as a genetic reference for fungal differentiation and secondary metabolism (4). The human pathogens Aspergillus fumigatus and Aspergillus flavus can cause pulmonary aspergillosis (5) or food spoilage by aflatoxin secretion on cereals and legumes (6). Invasive aspergillosis causes worldwide around 200,000 cases in humans per year, with limited availability of antifungal drugs to treat it (7).

Drug target search includes components of the ubiquitin-proteasome system (UPS), which is the prevailing conserved cellular protein destruction pathway in eukaryotes (8–11). Proteasome-targeted proteins are posttranslationally modified with ubiquitin mediated by E3 cullin-RING-ligases (CRLs) (12). The most common group of CRLs is the Skp1-Cul1-Fbx (SCF) complex, where Skp1 (S-phase kinase-associated protein 1) is the adaptor between the cullin scaffold and the Fbx (F-box) substrate receptor (13). SCFs are activated by the covalent modification of a lysine residue of cullin with the ubiquitin-like protein Nedd8 (neural precursor cell expressed, developmentally downregulated 8) (14). SCFs have to be disassembled and reassembled with different F-box proteins carrying different substrates in order to provide a broad substrate range (15). The exchange of F-box receptor units requires the interplay between the COP9 signalosome (CSN) deneddylase and the substrate receptor exchange factor Cand1 (Cullin-associated-Nedd8-dissociated protein 1) (16–18). CSN inactivates the SCF by removing Nedd8 from cullins (19, 20). Cand1 sequesters the cullin by blocking the Nedd8 binding site with the N-terminal domain. Cand1’s β-hairpin in helix B25 of the C-terminal domain interferes with the Skp1-adaptor binding site (21). The 120-kDa Cand1 is ubiquitously found in eukaryotes, where Cand1 is mostly encoded by a single gene. A. nidulans and other fungi of the class Eurotiomycetes possess at least two *candA* genes transcribed in opposite directions and are separated by five open reading frames. The encoded two subunits form a complex and fulfill the same molecular function as a single subunit Cand1, suggesting that an originally fused gene was split by rearrangement during evolution in the Eurotiomycetes (22). Accordingly, the human Cand1 counterpart of A. nidulans includes CandA-N and CandA-C proteins in a single polypeptide. In this study, a third A. nidulans CandA subunit was identified which has no counterpart in human Cand1. This newly identified *candA-C1* gene is located 269 bp upstream of *candA-C.* The human pathogen A. fumigatus found a different solution to cope with the split Cand proteins. It carries a fused gene where CandA-C1 is encoded by an additional exon, resulting in a 190-amino-acid N-terminal extension (NTE) of CanA. We found that an *Aspergillus*-specific CandA-C1, which is essential for E3 SCF activity, fungal growth, and development, was required to allow splitting of CandA in *Aspergillus* species. This resulted in a trimeric CandA complex in A. nidulans and a corresponding N-terminally extended dimeric complex in A. fumigatus. Antimycotic drug development aims to control fungal spreading. A. nidulans CandA-C1 and A. fumigatus CanA could serve as targets to specifically control the growth and spread of aspergilli to support human health and preserve agricultural products.

## RESULTS

### *Aspergillus* spp. possess an additional *candA* sequence as extension or separate gene.

**FIG 1:**
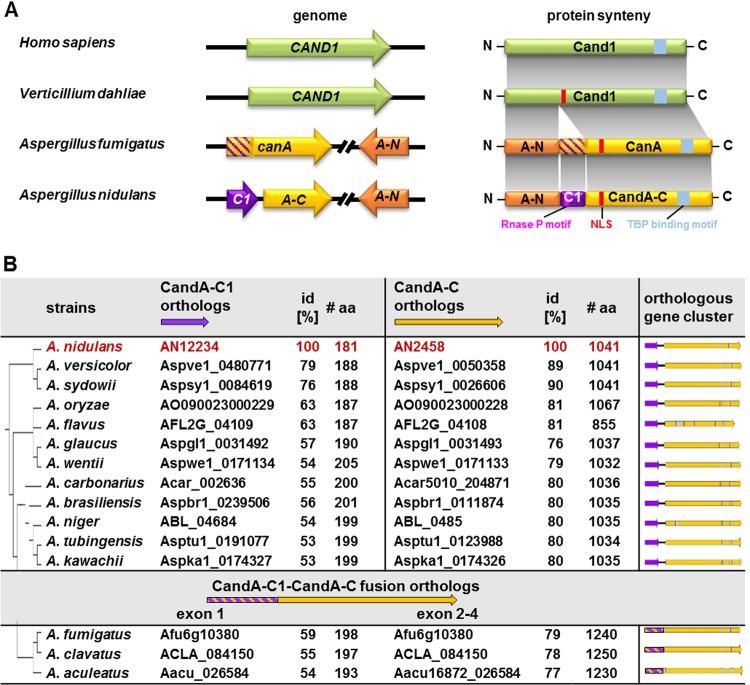
Comparison of single and split Cand proteins. (A) Comparison of human, Verticillium dahliae, Aspergillus fumigatus, and A. nidulans *candA* genes and encoding proteins. Human (Homo sapiens) genes and numerous fungal genes, such as those of V. dahliae, express a single Cand1 protein. The counterpart of human or fungal Cand1 is split in aspergilli in an N-terminal CandA-N/CanA-N (A-N) and C-terminal CandA-C/CanA part encoded by separate genes. Aspergilli require an additional Cand polypeptide CandA-C1 (A-C1) which is not present in humans. The CandA-C1 polypeptide represents the N-terminal part of the CanA protein of A. fumigatus but is encoded by a separate *candA-C1* gene (546 bp, 181 amino acids [aa], 19 kDa) in A. nidulans, which is located next and upstream to *candA-C* (3,254 bp, 1,041 aa, 113.5 kDa), which is separated from *candA-N* (1,055 bp, 313 aa, 33.6 kDa) by five open reading frames. CandA-C1 has an RNase P Rpr2/Rpp21 motif (pink), and V. dahliae Cand1 and *Aspergillus* CanA/CandA-C have an NLS (red) and a TATA binding protein (TBP) interaction motif (blue), which is conserved in all eukaryotic CandA C-terminal ends. H. sapiens Cand1, UniProt ID Q86VP6-1; V. dahliae Cand1, MycoCosm ID VDAG_05065T0; A. fumigatus CanA, UniProt ID Q4WMC6, and CanA-N, UniProt ID Q4WMC0; A. nidulans CandA-N, UniProt ID C8VP82; and CandA-C1, AspGD/FungiDB ID AN12234, CandA-C, UniProt ID Q5BAH2). (B) Comparative analysis of CandA-C1 and CandA-C orthologs in different aspergilli. The protein sequence of A. nidulans CandA-C1 (AN12234) was compared to those of different *Aspergillus* spp. by a BLASTp search in the Joint Genome Institute (JGI) MycoCosm genome portal. Genomic clusters of orthologs of separated *candA-C1* (purple) followed by intergenic ORF (iORF; black line) and downstream-located *candA-C* (yellow) genes are depicted. Three corresponding fused genes with the CandA-C1-like domain marked in purple-yellow stripes followed by an intron (gray) instead of the iORF are depicted in the bottom. Phylogenetic relationships are based on protein identities of the CandA-C1 orthologs in the genus *Aspergillus,* similar to relations described by de Vries et al. (2). id, protein identity.

The conserved eukaryotic Cand1/A protein functions as a substrate receptor exchange factor for cullin-RING ligases. Most organisms encode a single Cand gene. *Aspergillus* spp. are an exception and carry at least two genes coding in A. nidulans for CandA-N and CandA-C (22). Both genes are separated in most aspergilli by five genes coding for putative proteins, including septation-associated SepK, vacuolar biosynthesis-related Pep5/Vps11, or chitin deacetylase (Fig. 1A; see also Fig. S1A in the supplemental material). Cand1/A proteins have an armadillo-type fold typical of HEAT repeat proteins (21, 23, 24). CandA-C and CanA carry N-terminal nuclear localization signal (NLS) sequences (RKRRR) (22). A. fumigatus CanA carries an NTE encoded in exon-1 that corresponds to the deduced protein encoded by A. nidulans AN12234, which we named CandA-C1. This gene is located only 269 bp upstream of *candA-C* and was not considered before to encode a CandA subunit. CandA-C1, as well as A. fumigatus CanA NTE, have an N-terminal RNase P Rpr2/Rpp21 domain motif and, presumably, disordered C termini (Fig. 1A). This indicates that two A. fumigatus CanA proteins correspond to three A. nidulans CandA proteins, with 59% identity between CandA-C1 and CanA-NTE and 79% identity of CandA-C with full-length CanA. The two N-terminal orthologs share 77% protein identity.

10.1128/mBio.01094-19.1 FIG S1 The genomic environment of the *candA* genes within aspergilli. (A) Cartoon representation of the genetic region of *candA* genes of A. nidulans. The FungiDB synteny tool (1) was used for the analysis of *candA* localization in different *Aspergillus* species. The A. nidulans *candA* genomic locus was used as the basis for this search; the localization of *candA-C1* is indicated with purple arrows, *candA-C* is indicated with yellow arrows, and *candA-N* is indicated with orange arrows. Orthologous genes can also be tracked by continuous shading. Genes on the sense strand are depicted in blue and of the antisense strand in red. Species with a star indicate that they are annotated with five conserved open reading frames between *candA-C* and *candA-N* gene loci, which code for SepK (AN2459), AN2460 (uncharacterized), AN12235 (Pep5/Vps11 ortholog in S. cerevisiae), AN12236 (uncharacterized), and a putative chitin deacetylase (AN10309). CandA-C is the orthologous protein to A. fumigatus CanA, and a BLAST search revealed that CandA-N of A. nidulans corresponds in A. fumigatus to Afu6g10440 that was named CanA-N. *Ani*, A. nidulans; *Ave*, A. versicolor; *Asy, A. syndowii; Aor, A. oryzae; Afl*, A. flavus; *Agl, A. glaucus; Awe, A. wentii; Aca, A. carbonarius; Abr, A. brasiliensis; Atu*, A. tubingensis; *Afu*, A. fumigatus*; Acl, A. clavatus; Aac, A. acuelatus*. (B) The iORF contains the *candA-C* promoter and *candA-C1* terminator. Shown is the scheme of A. nidulans *candA* locus with labeled promoter (P), terminator (T), start codons (ATG) and stop codons (TGA or TAA). Continuous gray lines indicate the respective deletion strain, and broken lines show the resulting gene expression defect. (C) Phenotypes of a 5-day-old asexually developed A. nidulans wild-type strain compared Δ*candA-C1*, ^ΔATG^*candA-C1, candA-C1* complementation, Δ*iORF*, and Δ*candA-C1/iORF* mutant strains. The ATG deletion strain has the same growth defect as the Δ*candA-C1* mutant strain. A similar growth defect was observed for the Δ*iORF* and Δ*candA-C1/iORF* mutant strains, which additionally showed altered secondary metabolism (scale bars = 100 μm). Download FIG S1, TIF file, 0.7 MB. Copyright © 2019 Köhler et al. 2019 Köhler et al. This content is distributed under the terms of the Creative Commons Attribution 4.0 International license.

Bioinformatic analysis revealed that a majority of the 12 Aspergillus species carry the same two separated adjacent CandA-C1- and CandA-C-encoding genes as A. nidulans. Only A. fumigatus, Aspergillus clavatus, and Aspergillus aculeatus are annotated as expressing deduced fusion proteins with similar lengths and intron distributions (2) (Fig. 1B). These results suggest that *Aspergillus*-specific CandA-C1 could be linked to the conserved CandA protein family, mostly as an independent protein but also in some species as an N-terminal domain fusion to CandA-C. The surrounding positioning of homologous genes is conserved, indicating a common ancestor of all *Aspergillus* spp. which presumably had encountered a specific rearrangement of *candA* genes during evolution, which has not yet been described in other fungi.

### A. fumigatus expresses a single *canA* gene, and A. nidulans expresses two separate transcripts, *candA-C1* and *candA-C*.

**FIG 2:**
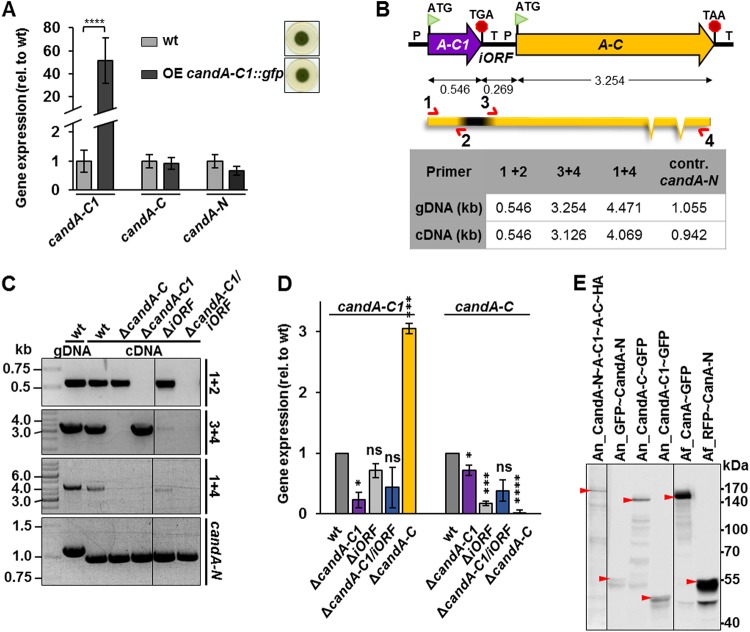
The intergenic ORF (iORF) contains the terminator for *candA-C1* and promoter for *candA-C* expression in A. nidulans. (A) Quantitative real-time PCR (qRT-PCR) measurements show the gene expression of *candA-C1, candA-C*, and *candA-N* in wild type (wt) compared to the overexpression (OE) *candA-C1*::*gfp* mutant strain. *candA-C1*::*gfp* is significantly overexpressed compared to wild-type expression but does not influence the transcription levels of *candA-C* or *candA-N* (****, *P ≤ *0.0001; *n* = 3). rel., relative. (B) cDNA amplification assay showing different PCR setups used to amplify *candA-C1* (primer 1 + 2 [oAMK120/121]), *candA-C* (primer 3 + 4 [oAMK03b/04b]), and *candA-C1*::*candA-C* (primer 1 + 4 [oAMK120/04b]). The table shows expected sizes in kilobase pairs from gDNA or cDNA. The amplification of *candA-N*, which contains two introns, was used as control to exclude gDNA contamination in cDNA samples (primer oAMK01/02). (C) Agarose gel pictures of PCR products are depicted, and sizes are indicated. (D) qRT-PCR experiments show the expression levels after 20 h of vegetative growth of *candA-C1* and *candA-C* in the wild type compared to the *candA-C1, iORF, candA-C1/iORF*, and *candA-C* deletion mutants. The expression of *candA-C1* is significantly upregulated in a Δ*candA-C* mutant sample, and *candA-C* is significantly downregulated when the *iORF* is deleted (*, *P ≤ *0.05; ***, *P ≤ *0.001; ****, *P ≤ *0.0001; *n* = 3, except for Δ*candA-C1/iORF* mutant, *n* = 2). (E) Western hybridization of 20-h-old vegetative A. nidulans and A. fumigatus CandA/CanA subunits probed with anti-HA (left panel), anti-GFP (middle panels), and anti-GFP and anti-RFP (right panels) antibodies shows that A. fumigatus CanA-GFP (163 kDa) and A. nidulans CandA-C-GFP (142 kDa) exhibit different molecular weights. GFP-CandA-N and RFP-CanA-N have the same weight (59 kDa) and show a double band. CandA-C1-GFP runs at 47 kDa, and a fusion of CandA-N∼A-C1∼A-C-HA runs at 170 kDa.

The gene expression levels of *candA-C1, candA-C*, and *candA-N* in A. nidulans were compared by quantitative real-time PCRs (qRT-PCRs) from the wild type and a strain overexpressing *candA-C1*::*gfp* by the nitrate promoter. Fifty-fold overexpression of *candA-C1* neither changed the fungal phenotype caused by overexpression of *candA-C1*::*gfp* nor altered the expression levels of *candA-C* and *candA-N* (Fig. 2A). This indicates that *candA-C1* and c*andA-C* are separate genes, and the 269-bp intergenic-open reading frame (iORF) region between *candA-C1* and *candA-C* ORFs should include a terminator for *candA-C1* and a promoter for the *candA-C* gene. RNA sequencing (RNA-seq) data deposited in FungiDB showed expressed sequences of both ORFs, with fewer *candA-C1* fragments per kilobase of exon model per million mapped reads than *candA-C*, supporting differences in expression between the two genes.

The ORFs of *candA-C1, candA-C*, and a putative fusion of *candA-C1*::*candA-C* were amplified from wild-type genomic DNA (gDNA) and complementary DNA (cDNA), as well as of mutant strain cDNA, to examine the transcripts of the iORF region (Fig. 2B). Specific PCR products for *candA-C1* were obtained from wild-type c/gDNA, Δ*candA-C* mutant, and Δ*iORF* mutant strains (Fig. 2C). Except for a faint signal of *candA-C* amplification in the Δ*iORF* mutant strain, a significant PCR product of *candA-C* was only observed from PCRs on wild-type c/gDNA and when *candA-C1* was deleted. This corresponds to the significant downregulation of *candA-C* expression when the iORF was missing in qRT-PCR, supporting the idea that the iORF includes the promoter sequence for *candA-C* (Fig. 2D). The *candA-C1* gene expression was 3-fold increased in a *candA-C* deletion strain. These observations are consistent with the phenotypes of a strain lacking the *candA-C1* start codon, the Δ*iORF* mutant, and the Δ*candA-C1/iORF* mutant strain (Fig. 2C and S1B and C). A combined *candA-C1*::*candA-C* transcript was amplified and might be an antisense transcript, which is supported by FungiDB RNA-seq data. The A. fumigatus CanA-green fluorescent protein (CanA-GFP) (163 kDa) and A. nidulans CandA-C-GFP (142 kDa) exhibited different molecular weights in Western hybridization, and the expression of a fused A. nidulans *candA* gene resulted in a 170-kDa protein, which correlates with the sum of the single subunits (Fig. 2E). These results demonstrate that *candA-C1* and *candA-C* are separate genes in A. nidulans, with an iORF containing *candA-C1* terminator and *candA-C* promoter sequences, whereas the orthologous A. fumigatus CanA combines both peptides, including an NTE corresponding to CandA-C1.

### CandA-C1 interacts with CandA-C and CandA-N.

**FIG 3:**
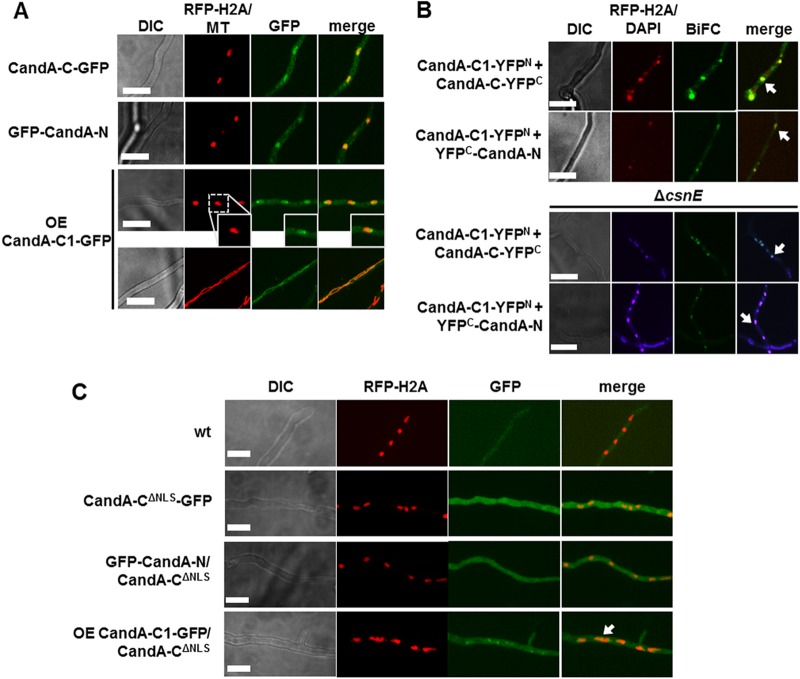
A. nidulans CandA-C1 interacts with CandA-C and CandA-N. (A) Localization of native CandA-C-GFP, GFP-CandA-N, and overexpression (OE) CandA-C1-GFP proteins. All three CandA proteins colocalize with monomeric RFP (mRFP)-tagged histone H2A (RFP-H2A). CandA-C1-GFP is also localized to nucleoli and colocalizes with MitoTracker Red (MT) that stains mitochondria. (B) Bimolecular fluorescence complementation (BiFC) microscopy of CandA-C1 with CandA-C and CandA-N in a wild-type background as well as in the *csnE* deletion strain. BiFC signals are visible in green and localized to the nuclei, which are stained with H2A-RFP or 4′,6-diamidino-2-phenylindole (DAPI). DIC, differential interference contrast. (C) Nuclear localization of CandA-C and CandA-N is dependent on CandA-C NLS, as both CandA proteins are absent from RFP-H2A-stained nuclei when the NLS is deleted. OE CandA-C1-GFP is localized to the nuclei and nucleoli (white arrows indicate colocalization to the nuclei; scale bars = 10 μm).

Localization of GFP-tagged CandA proteins from A. nidulans by fluorescence microscopy revealed that GFP-CandA-N and CandA-C-GFP are mainly localized to the cytosol and nuclei. CandA-C1-GFP is localized to the nuclei, nucleoli, cytosol, and, presumably, mitochondria (Fig. 3A). Bimolecular fluorescence complementation (BiFC) microscopy showed that CandA-C1 interacts with CandA-N and CandA-C in the nuclei and sometimes in mitochondria in wild-type and cullin deneddylation-deficient *csnE* deletion strains (Fig. 3B and S2A and B).

10.1128/mBio.01094-19.2 FIG S2 CandA-C1 nuclear localization is independent on CandA-N and CandA-C. (A) BiFC control strains expressing one-half of yellow fluorescent protein (YFP) without fused protein and the other half of YFP tagged to the protein of interest show no signals of interaction. (B) BiFC signals of the interaction of CandA-C1 with CandA-C and CandA-N colocalized to mitochondria, which were labeled with MitoTracker Red (MT). The surface view was generated from z-stack pictures depicting the BiFC signal in green and the mitochondria in red. Nuclei are stained with histone H2A-labeled red fluorescent protein (RFP-H2A). (C) OE CandA-C1-GFP is localized to nuclei and nucleoli in Δ*candA-N*, Δ*candA-C* and Δ*candA-N/C* mutant strains. CandA-N and CandA-C are localized to nuclei in a Δ*candA-C1* mutant strain (white arrows indicate colocalization to nuclei; scale bars = 10 μm). Download FIG S2, TIF file, 0.6 MB. Copyright © 2019 Köhler et al. 2019 Köhler et al. This content is distributed under the terms of the Creative Commons Attribution 4.0 International license.

The CandA-C backpacks CandA-N into the nucleus (23), and its NLS (RKRRR) at amino acid positions 138 to 142 is required for CandA-C and CandA-N nuclear transport. Nuclear localization of CandA-C1 is independent of the CandA-C NLS and also of the presence of CandA-C or CandA-N. Conversely, CandA-N and CandA-C are also nuclear in the absence of CandA-C1 (Fig. 3C and S2C). Therefore, CandA-C1 and CandA-C travel independently into the nucleus, and only the nuclear transport of CandA-N is dependent on CandA-C.

### A. nidulans CandA pulls only CulA, whereas A. fumigatus CanA recruits CulA and CulC.

**FIG 4:**
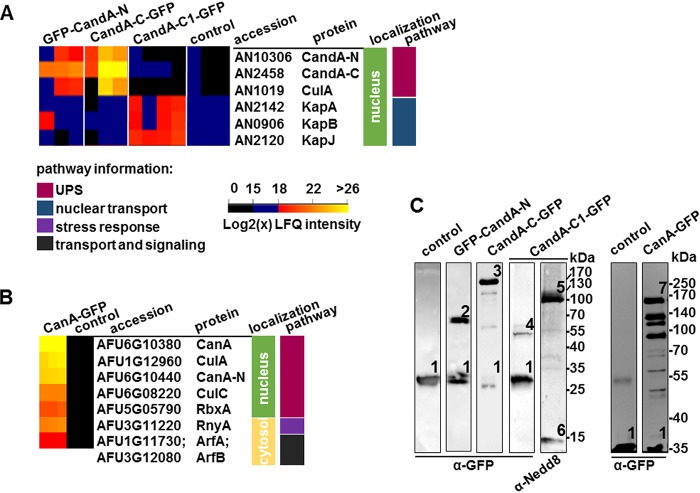
GFP-pulldown coupled to LC-MS analysis of A. nidulans and A. fumigatus CandA proteins. (A) Comparison of putative interaction partners of CandA-C and CandA-N with CandA-C1 from pulldown experiments. An excerpt of the heatmap, generated with Perseus (version 1.6.0.7), depicts label free-quantification (LFQ) intensities of three biological replicates of GFP control, CandA-N, CandA-C, and four replicates of CandA-C1. Log_2_(x) LFQ intensities range from 0 to 15, not considered to be identified (black); 15 to 18, low intensity (blue); and 18 to 26/32, low to high LFQ intensities (gradient from red to orange to yellow). Colored bars indicate cellular localization, based on KEGG and UniProt databases (green, nucleus; light yellow, cytosol). The molecular pathway of putative interaction partners is labeled as follows: berry, ubiquitin-proteasome system (UPS); dark blue, nuclear transport; purple, stress response; and dark gray, transport and signaling. (B) Heatmap of identified proteins from A. fumigatus CanA-GFP pulldown compared to overexpression GFP control strain. (C) Western hybridization of pulldown elution samples probed with anti-GFP antibody, which shows free GFP (1), GFP-CandA-N (2), CandA-C-GFP (3), CandA-C1-GFP (4), and CanA-GFP (7). The elution sample of CandA-C1-GFP was reprobed with anti-Nedd8 antibody, which highlights neddylated cullins (5) and free Nedd8 (6).

GFP-pulldown experiments from fungal cell extracts of A. nidulans strains expressing functional GFP-CandA-N, CandA-C-GFP (native promoter), and CandA-C1-GFP (overexpressing) and from A. fumigatus CanA-GFP (native promoter) were performed to compare the complexes from the two fungi. MaxQuant analysis of the liquid chromatography-mass spectrometry (LC-MS) data yielded the identification of about 2,000 proteins. Downstream processing and filtering of the data with Perseus revealed 51 significantly enriched candidates (Table S1). Peptides of CandA-N and CandA-C showed highest log_2_(x) label-free quantification (LFQ) intensities in CandA-C-GFP and GFP-CandA-N pulldowns. CulA was identified as among the best candidates, whereas CulC or CulD could not be identified, indicating that A. nidulans CandA is specific for CulA-containing CRLs. A. fumigatus CanA-GFP pulled CanA-N, both CulA and CulC, and RbxA (Fig. 4A and B). Western hybridization of the CandA-C1-GFP elution fraction using anti-Nedd8 antibody revealed signals of interacting neddylated cullins, as well as free Nedd8, whereas cullins were not identified in LC-MS data analysis of CandA-C1-GFP (Fig. 4C). Western hybridization and MS LFQ intensities corroborate the idea that CandA-C1 is part of a trimeric CandA complex and might exhibit additional cellular functions in A. nidulans independently of CandA-N or CandA-C.

10.1128/mBio.01094-19.3 FIG S3 CandA-C1, CandA-C, and CandA-N support conidiation, and CandA-C1 is required for growth. (A) Asexual phenotypes of 5-day-old A. nidulans wild type and *candA-C1, candA-C*, and *candA-N* complementation strains (scale bars = 100 μm). (B) Quantitative real-time PCR (qRT-PCR) measurements of *candA-C1* expression levels in overexpression strains relative to the wild type. Overexpression levels of *candA-C1* are around 50 times higher in mutant strains than in the wild type (*n* = 2). (C) Overexpression *candA-C1* increases conidiospore production but reduces colony size in the absence of *candA-C* and *candA-N/A-C* in A. nidulans after five days of incubation at 37°C in the light. Overexpression of *candA-C1* appears like wild type and does not alter the phenotype when expressed in a Δ*candA-N* background strain. (D) Quantification of colony diameter and conidiospores after five days of asexual development (*n* = 3, error bars represent the standard error of the mean [SEM]). (E) Plates incubated in the dark and with limited oxygen supply for seven days for sexual development. *candA* deletion mutants with overexpressed *candA-C1* are unable to form cleistothecia, whereas the overexpression in the wild-type background shows wild-type-like cleistothecial production (scale bars = 100 μm; c = cleistothecia). (F) Phenotypes of 14-day-old A. fumigatus wild-type and *canA* mutant strains incubated at 37°C. (G) Mitochondria of Δ*candA* deletion strains are fragmented. Confocal fluorescence microscopy was performed with wild-type (wt), *candA* deletion, and overexpression *candA-C*::*gfp* (OE *candA-C1*::*gfp)* mutant strains after 10 and 24 h of incubation in liquid MM supplemented with *para*-aminobenzoic acid (PABA) at 37°C. Hyphae were stained with MitoTracker Red prior to taking pictures. Whereas wt and OE strains show long mitochondrial filaments, the Δ*candA-C1* mutant has fragmented mitochondria already after 10 hours. The Δ*candA-C* and Δ*candA-N* mutants show less fragmentation which increases over time (scale bars = 10 μm; −, no fragmentation; +, fragmentation). (H) Thin-layer chromatography indicates emerimidine production in the *candA-C* and *candA-N* deletion strains. Ethyl acetate extracts of A. nidulans wild-type and *candA-C1, candA-C*, and *candA-N* deletion strains from seven days of asexual development (left) and sexual development (right) at 366 nm with 1,000-ms exposure show a blue band at R*_f_* = 0.43, which correlates to emerimidine (2). Download FIG S3, TIF file, 1.2 MB. Copyright © 2019 Köhler et al. 2019 Köhler et al. This content is distributed under the terms of the Creative Commons Attribution 4.0 International license.

10.1128/mBio.01094-19.4 TABLE S1 Identified proteins in A. nidulans CandA-N, CandA-C, and CandA-C1 pulldowns. Detailed information about identified proteins from the heatmap in Fig. 4A as a result of filtering. Download Table S1, DOCX file, 0.1 MB. Copyright © 2019 Köhler et al. 2019 Köhler et al. This content is distributed under the terms of the Creative Commons Attribution 4.0 International license.

### A. nidulans CandA is required for SCF activation.

**FIG 5:**
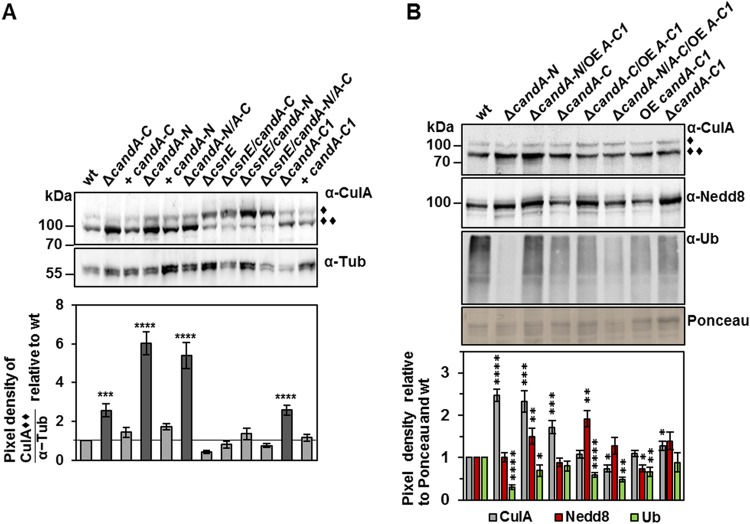
CandA is required for CulA neddylation in A. nidulans. Western hybridization was performed with crude extracts from 20-h-grown vegetative mycelium. (A) Western hybridization probed with anti-CulA antibody to observe CulA neddylation ratios. In the wild type, most CulA is deneddylated (∼96 kDa, ◆◆), which is different from a Δ*csnE* strain, which is defective in cullin deneddylation and has most CulA bound to Nedd8 (∼106 kDa, ◆). In the Δ*candA* and Δ*candA-C1* deletion strains, deneddylated CulA accumulates. Double and triple deletions of *csnE, candA-N*, and/or *candA-C* show an accumulation of neddylated CulA as observed for the Δ*csnE* mutant. Signals were quantified with pixel density measurements using BIO1D software (Peqlab) for total 12 replicates (three biological replicates each with four technical replicates). Tubulin (Tub) served as a loading control. (B) Western hybridization probed with anti-CulA (gray), anti-Nedd8 (red), and anti-ubiquitin (α-Ub) (green) antibodies. Ponceau served as loading control. The Δ*candA* mutant strains were compared to *candA-N* and *candA-C* deletion strains overexpressing *candA-C1*. The pixel density ratio was determined with the BIO1D software (Peqlab), quantified against Ponceau, and normalized to wild-type signals. CulA and Ub used three biological with three technical replicates each, and Nedd8 used four biological with three technical replicates each; error bars represent the standard error of the mean. *, *P ≤ *0.05; **, *P ≤ *0.01; ***, *P ≤ *0.001; ****, *P ≤ *0.0001.

The CulA neddylation status in A. nidulans *candA* deletion strains was investigated with *in vitro* deneddylation assays to analyze whether all three subunits have an impact on cullin neddylation. Deneddylated CulA is visualized as lower and neddylated CulA as higher migrating signals in Western hybridization experiments (17, 25). Larger amounts of deneddylated CulA were visible in the Δ*candA-C1*, Δ*candA-C*, Δ*candA-N*, and Δ*candA-N/A-C* mutant strains than in the wild type, with the most pronounced 2-fold-higher effect in the absence of CandA-N. Fewer ubiquitinated proteins were observed in all *candA* deletion strains and are presumably a direct consequence of increased inactive CulA. Double- and triple-deletion strains of *candA* with deneddylase-deficient *csnE* showed increased neddylated CulA levels, like those of a *csnE* single-deletion strain. The total neddylated cullins were similar in the Δ*candA* mutant strains (Fig. 5A and B). Therefore, all three CandA subunits contribute to the accurate ratio of neddylated relative to deneddylated CulA within the fungal cell.

The effect of CandA-C1 on SCF activity was investigated using the *candA-C1*::*gfp* overexpression strain in wild-type and Δ*candA-N* and Δ*candA-C* mutant backgrounds. The amount of deneddylated CulA was reduced to wild-type levels when *candA-C1*::*gfp* was overexpressed in the Δ*candA-C* or Δ*candA-N/A-C* mutant, but nothing changed in the Δ*candA-N* mutant strain. The overexpression of *candA-C1* alone did not have any effect on deneddylated CulA. Elevated *candA-C1*::*gfp* expression increased the total neddylated cullin levels of *candA-N/A-C* single- and double-deletion strains (Fig. 5B). CandA-C1 overexpression can therefore only rescue defects in the CulA neddylation cycle caused by the absence of CandA-C, but not of CandA-N, and it increases the total neddylated cullin pool. These data further suggest that after the initial deneddylation of CRLs by CSN and subsequent CandA-mediated CRL disassembly, CandA has an additional novel function. It is also required to initiate a new cycle of neddylation and activation of CRLs with another substrate receptor. Therefore, CRL activity is not only dependent on a functional CSN but also on the interplay of CandA-N, CandA-C1, and CandA-C.

### CandA-C1 and CanA promote growth and development in A. nidulans and A. fumigatus.

**FIG 6:**
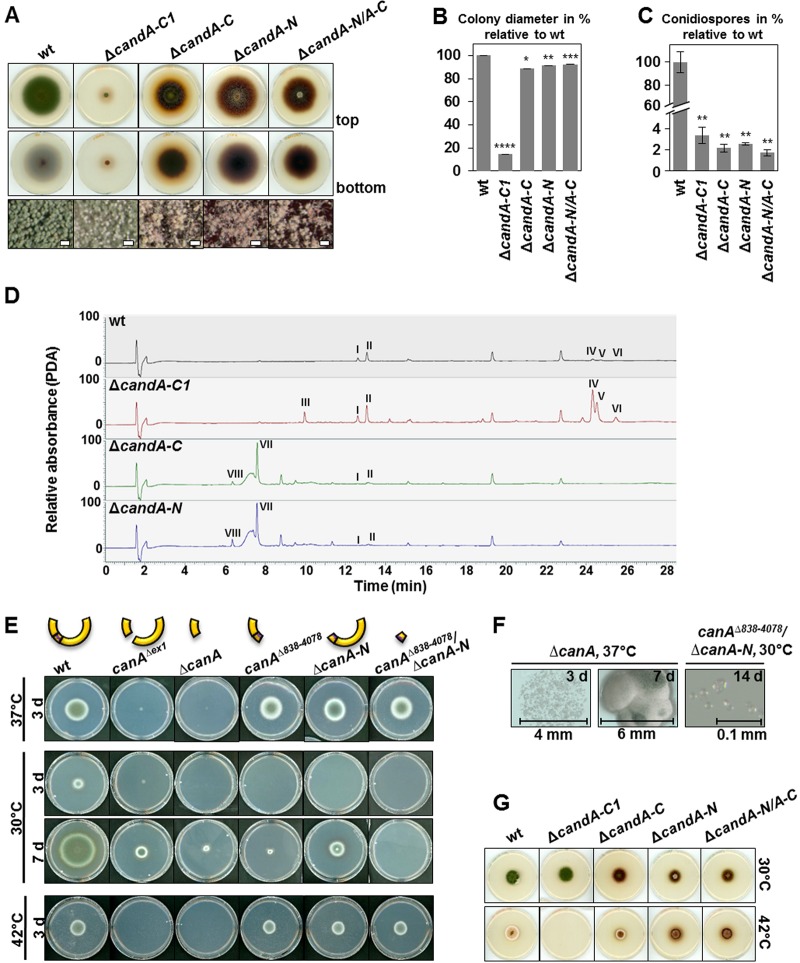
A. nidulans CandA-C1 and A. fumigatus CanA are required for growth and asexual development. (A) A. nidulans conidia (4 × 10^3^) were point inoculated on solid minimal medium supplemented with *para*-aminobenzoic acid and incubated at 37°C in light for 5 days. Pictures were taken from the top and bottom views of the plate. Binocular pictures show asexual spores (scale bars = 100 μm). (B and C) Quantification of colony diameter (B) and amount of spores after 5 days of asexual growth (C) in percentage (%) relative to the wild type. Error bars represent the standard error of the mean (SEM) (*n* = 3). (D) LC-MS combined with photodiode array detection (PDA) analysis of secondary metabolites extracted from 7-day-old asexually developed mycelium revealed differences in all tested strains. The wild type and Δ*candA-C1* mutant produce similar amounts of austinol (I) and dehydroaustinol (II). The Δ*candA-C1* mutant produces asperthecin (III) and greater amounts of emericellin (IV) and shamixanthone/epishamixanthone (V and VI) than does the wild type. Metabolites III, IV, V, and VI were absent in the Δ*candA-C* and Δ*candA-N* mutants, but both produced cichorine (VII) and a metabolite (VIII) with high-resolution–electrospray ionization–MS (HR-ESI-MS) at *m/z* 210.0761 [M+H]^+^ (calculated for C_10_H_12_NO_4_, *m/z* 210.0766). (E) A. fumigatus conidia (4 × 10^3^) were point inoculated on solid modified minimal medium and incubated at 37°C, 30°C, and 42°C for 3 to 7 days. The Δ*canA* mutant strains are growth defective at all tested temperatures. (F) Micrographs of Δ*canA* mutant colonies after 3 and 7 days of growth at 37°C, and micrograph of Δ*canA^838-4078^/*Δ*canA-N* mutant after 14 days at 30°C shows colorless conidia, which did not germinate. (G) A. nidulans conidia (4 × 10^3^) were point inoculated on solid minimal medium supplemented with *para*-aminobenzoic acid and incubated at 30°C and 42°C in light for 5 days. The *candA* mutant strains grew like the wild type at 30°C and 42°C, except for the Δ*candA-C1* mutant, which grew better at 30°C than at 37°C and was unable to germinate at 42°C.

Single- and double-deletion A. nidulans *candA-N* and *candA-C* mutant strains showed colony diameters similar to those of the wild type but produced fewer conidia. The hyphae and surrounding media were colored dark red-brown, indicating an altered secondary metabolism (Fig. 6A to D and S3A). Analysis of secondary metabolites from asexual development by LC-MS revealed that both strains produce cichorine (VII) that was hardly detectable in the wild type or Δ*candA-C1* mutant. Peak VIII, present in the Δ*candA-C* and Δ*candA-N* mutants, corresponds to an unknown metabolite with mass of *m/z* 210.0761 [M+H]^+^ and deduced molecular formula C_10_H_11_NO_4_. Austinol (I), dehydroaustinol (II), asperthecin (III), emericellin (IV), and shamixanthone/epishamixanthone (V and VI) were increased in the Δ*candA-C1* mutant strain in comparison to the wild type, which does not produce detectable asperthecin (Fig. 6D and Data Set S1).

10.1128/mBio.01094-19.10 DATA SET S1 Extracted ion chromatogram (EIC), MS^2^, and UV-Vis spectra of identified secondary metabolites from asexual and sexual development of A. nidulans wild-type (wt) and *candA* deletion strains. Download Data Set S1, PDF file, 0.7 MB. Copyright © 2019 Köhler et al. 2019 Köhler et al. This content is distributed under the terms of the Creative Commons Attribution 4.0 International license.

The Δ*candA-C1* mutant grew 6 times slower but produced 1.5 to 2 times more conidia than the *candA-N/A-C* single- and double-deletion strains, which was still 30 times less than the amount of spores produced by the wild type (Fig. 6A to C). Conidial formation was increased, but the colony radius decreased when *candA-C1* was overexpressed in the Δ*candA-C* or Δ*candA-N/A-C* mutant but not in the Δ*candA-N* mutant strain, suggesting that *candA-C1* expression can rescue conidiation defects caused by the loss of *candA-C* (Fig. S3B to D). CandA-C1 therefore has a distinct additional cellular function and promotes vegetative growth and conidial formation.

The A. fumigatus *canA* and *canA*^Δ^*^exon1^* deletion mutant strains were delayed in germination and showed a significant growth retardation and adjourned development, suggesting that CanA promotes spore germination and colony growth. Single deletions of Δ*canA-N* or of the *canA* domain (bp 838 to 4078) corresponding to A. nidulans *candA-C*, as well as the double-deletion strain, showed a delay in conidial formation that was visible as a white halo surrounding the colony. CandA/CanA supports growth at different temperatures, and the full A. fumigatus CanA complex is required for spore germination at 30°C (Fig. 6E to G and S3F).

**FIG 7:**
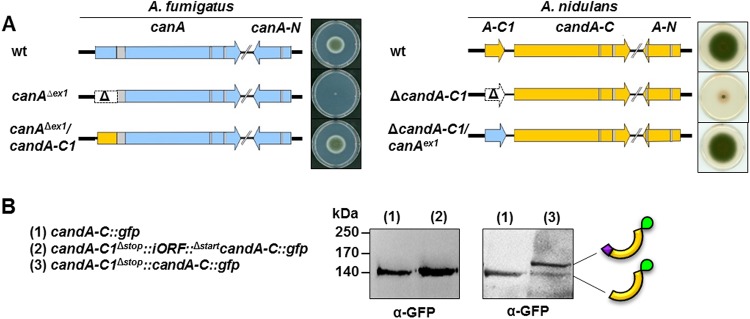
A. fumigatus and A. nidulans gene orthologs for *candA-C1* are interchangeable with each other. (A) Genome map of A. fumigatus (blue) wild-type *canA* and *canA-N*, a mutant strain carrying a *canA*^exon1^ deletion (Δ), and a mutant strain with the replacement of *canA^exon1^* with the A. nidulans *candA-C1* sequence (yellow). Introns are colored in gray. Genome map of A. nidulans (yellow) wild-type *candA-C1, candA-C*, and *candA-N*, a mutant strain with *candA-C1* deletion (Δ), and a mutant strain where *candA-C1* is replaced with A. fumigatus *canA^exon1^* (blue). Solid modified minimal medium was point inoculated with 4 × 10^3^ conidia of A. fumigatus strains for 3 days at 37°C in darkness. Solid minimal medium was point inoculated with 4 × 10^3^ conidia of A. nidulans strains and incubated at 37°C with illumination for 5 days. (B) Western hybridization with anti-GFP antibody of A. nidulans protein crude extracts from a strain expressing CandA-C-GFP [140 kDa (1)] and two strains carrying *candA-C1*::*candA-C*::*gfp* fusion constructs with (2) and without (3) the iORF sequence. A CandA-C1–CandA-C–GFP fusion protein (161 kDa) was expressed from the construct without iORF also showing a signal for CandA-C-GFP.

The conservation of the function of *candA-C1* from both species was examined. The construction of an A. fumigatus strain with integrated An_*candA-C1* sequence into the genomic locus of *Af_canA^exon1^* and an A. nidulans strain with the *Af_canA^exon1^* sequence introduced into the genomic locus of *candA-C1* revealed that the two sequences are interchangeable (Fig. 7A). Generation of a CanA-like fusion protein in A. nidulans displayed a CandA-C1-CandA-C-GFP protein migrating at higher molecular weight than CandA-C-GFP (Fig. 7B). A comparison of A. nidulans with A. fumigatus *candA/canA* mutants revealed that CandA-C1 supports vegetative growth. The CandA orthologs have conserved functions in conidiation, although A. nidulans has a trimeric complex and A. fumigatus a dimeric complex.

### CandA is required for sexual development, and CandA-C1 coordinates secondary metabolite genes other than those encoding CandA-C and CandA-N.

**FIG 8:**
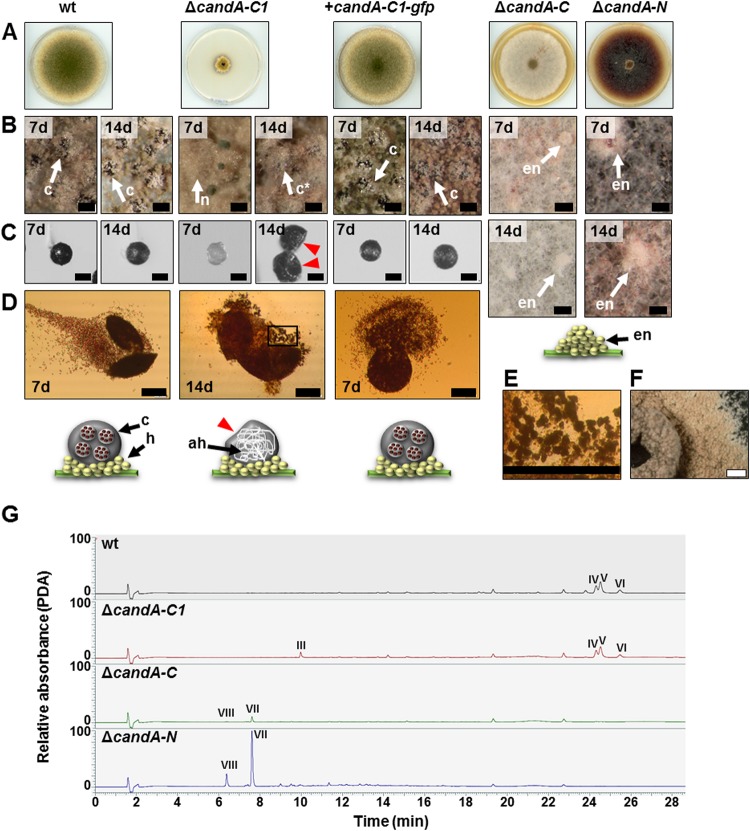
Ascospore formation is dependent on *candA-C1* in A. nidulans. Solid minimal medium was point inoculated with 4 × 10^3^ conidia and incubated in the dark and with limited oxygen supply for seven and 14 days. (A) Pictures of sexual phenotypes were taken after 7 days. (B) Micrograph pictures show cleistothecia (c) covered by Hülle cells (h) for wild-type (wt) and the *candA-C1* complementation strain. The *candA-C1* deletion strain has nests (n) after 7 days which develop to empty cleistothecia (c*) after 14 days. *candA-C* and *candA-N* deletion strains only produce early nests (en) but cannot undergo a complete sexual life cycle. (C) Micrograph pictures of Hülle cell-free cleistothecia. *candA-C1* has soft cleistothecia with dents, indicated with red arrows. (D and E) Microscopic pictures of squeezed cleistothecia never showed any mature ascospores for the *candA-C1* deletion strain (D) but did show ascogenous hyphae (ah) (E). (F) Closeup view of the Δ*candA-C1* mutant colony. Black scale bars = 100 μm; white scale bar = 1,000 μm. d, days. (G) LC-MS combined with photodiode array detection (PDA) analysis of secondary metabolites extracted from 7-day-old sexually developed mycelium revealed that the Δ*candA-C1* mutant produces asperthecin (III). The wild type and Δ*candA-C1* mutant produce similar amounts of emericellin (IV) and shamixanthone/epishamixanthone (V and VI). Small amounts of cichorine (VII) were detected in the Δ*candA-C* mutant, which were increased in the Δ*candA-N* mutant. The Δ*candA-C* and Δ*candA-N* mutants produced compound VIII with HR-ESI-MS at *m/z* 210.0761 [M+H]^+^ (calculated for C_10_H_12_NO_4_, *m/z* 210.0766).

The sexual life cycle of A. nidulans serves for overwintering of ascospores (26, 27). Hülle cells protect and nurse the early nests that develop to primordia and maturate in 7 days to fruiting bodies (cleistothecia) (28). Each cleistothecium contains several asci, with each harboring eight ascospores. Strains missing *candA-N* and *candA-C* are blocked in sexual development at the stadium of early nest production (22). The colony of the Δ*candA-C1* mutant strain was covered with yellowish Hülle cells forming nests after 7 days of development and produced a volcano-like phenotype by growing vertically with a hole in the middle of the colony. Nests of the Δ*candA-C1* mutant contained only primordia after 7 days, and sexual fruiting bodies with a moderately soft and fragile surface were present after 14 days, indicating a delayed sexual development. These cleistothecia did not contain any ascospores but did contain a complex network of ascogenous hyphae (Fig. 8A to F). Large amounts of CandA-C1-GFP were not sufficient to rescue cleistothecial formation in the *candA-C* and *candA-N* deletion strains (Fig. S3E). These data show that CandA-C and CandA-N are required for nest formation and primordial development, whereas CandA-C1 has a later function. CandA-C1 is required for stable cleistothecial wall formation and ascospore development.

Analysis of secondary metabolite production after 7 days of sexual development showed that Δ*candA-C* and Δ*candA-N* mutants produce cichorine and metabolite VIII, similar to what is observed in asexual development. Asperthecin, the red dye of cleistothecia, as well as emericellin and shamixanthone/epishamixanthone, were detected by LC-MS in the Δ*candA-C1* mutant strain (Fig. 8G and Data Set S1), indicating that CandA-C1 coordinates secondary metabolite genes other than those for CandA-C and CandA-N.

In summary, we demonstrate that a likely genomic rearrangement of a single fungal *candA* gene during evolution required an additional component which had to be integrated and resulted in changes in the subunit compositions of CandA in aspergilli. A. fumigatus and A. nidulans represent two different groups with different solutions, dimeric, which includes an N-terminal extension, versus a trimeric CandA complex including similar genetic information. The NTE found in A. fumigatus CanA corresponds to the separated single CandA-C1 subunit of A. nidulans. This trimeric CandA complex is required for CRL activity, supports asexual and sexual development, and thereby has influence on the secondary metabolism.

## DISCUSSION

**FIG 9:**
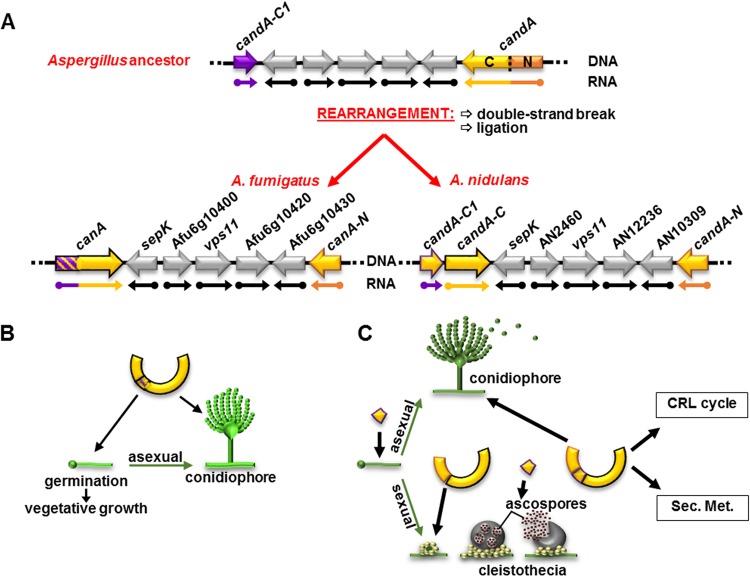
A trimeric CandA is required for growth, development, a coordinated secondary metabolism (Sec. Met.), and the CRL cycle in *Aspergillus* spp. (A) Scheme of a putative *Aspergillus* ancestor and DNA rearrangement of the *candA* loci. The ancestor of all *Aspergillus* spp. presumably had one gene containing sequence information of *candA-N* and *candA-C.* A DNA double-strand break, followed by ligation, has changed the position of *candA-C* five open reading frames upstream of *candA-N* and directly downstream of *candA-C1* in A. nidulans. In A. fumigatus, the *candA-C1-*like sequence fused to the rearranged *canA.* (B) A. fumigatus CanA N-terminal extension is essential for properly timed spore germination and vegetative growth. CanA and CanA-N promote germination and vegetative growth during low-temperature stress and promote conidiophore development. (C) A. nidulans CandA-C1 promotes germination and vegetative growth. CandA-N and CandA-C are essential for multicellular sexual fruiting bodies from the stage of early nest formation. CandA-C1 is required for properly timed cleistothecia formation and is essential for the development of ascospores. All three CandA proteins support the CRL cycle and conidiophore development. Furthermore, CandA contributes to secondary metabolism control.

The ubiquitin-proteasome pathway includes the dynamic interplay of the substrate receptor exchange factor CandA and three macromolecular multiprotein complexes, SCF E3 ubiquitin RING ligase, CSN deneddylase, and the 26S proteasome. The three ZOMES complexes CSN, proteasomal lid, and translation eukaryotic initiation factor 3 (eIF3) presumably have a common origin because they share similar subunits in a common architecture, with some variations in subunit compositions (29, 30, 64). Similarly, the subunit composition of the CSN antagonist Cand1/A is divergent in eukaryotes. The putative ancestor of all *Aspergillus* spp. might have had one *candA* gene encoding a single subunit CandA with N- and C-terminal domains corresponding to human Cand1. A. fumigatus is a representative of a group of species with a dimeric complex, which includes the N-terminal (CanA-N) and an extended C-terminal (CanA) part of human Cand1. A. nidulans represents a larger group of aspergilli which even form a trimeric CandA substrate receptor exchange factor complex, where the N-terminal extension of A. fumigatus CanA corresponds to the third subunit CandA-C1 in addition to the split CandA-N and CandA-C subunits, which we described earlier (22) (Fig. 9A). This represents an interesting example of evolutionary protein complex formation based on the splitting of one gene into two and combining the protein products with a polypeptide of an additional open reading frame providing additional functions, which is expressed as additional exon or as separate gene. The *candA-C1* gene was presumably a separate gene which encodes a putative RNase P subunit with an Rpr2/Rpp21 motif in the N-terminus that is also found in A. fumigatus CanA. RNase P is a RNA-protein complex (ribozyme) which can cleave RNAs, such as, 5′ precursors of tRNAs, and exists in different compositions of proteins and RNA, whereby A. nidulans encodes one nuclear and one mitochondrial RNA and seven associated proteins, including CandA-C1 (Table S2) (29, 30). It is currently elusive whether CandA-C1 is still part of a RNA complex. It is specific for Eurotiomycetes in the division of Ascomycota, whereas higher eukaryotes lack an ortholog. The current gene order of the *candA* genes could be caused by a DNA double-strand break and subsequent rearrangement. The position of the *candA-C* gene changed to a position downstream of *candA-C1.* The A. nidulans iORF includes a terminator of *candA-C1* and a *candA-C* promoter. It is elusive whether CandA-C1 was already functionally linked to CandA before the rearrangement or as result of reordering of genes. The consequences of the rearrangement of the A. nidulans *candA* genes are separate expression of *candA-C1, candA-C*, and *candA-N*. In A. fumigatus, the rearranged *canA* C-terminal sequence hijacked the upstream *candA-C1* gene, which resulted in a fused gene encoding a CanA protein with an N-terminal extension and a separate *canA-N* gene. The fusion of these genes might have facilitated the response to stress, like temperature, oxidative, or heavy-metal stress, and sterol-biosynthesis-inhibiting triazole fungicides (31–33). The different organization of *canA* genes in A. fumigatus could be due to selective pressure that maintains the diversity of pathogens to avoid detection by the host immune system or might correlate with the heterothallism and thereby limited recombination by the sexual life cycle (31, 34).

10.1128/mBio.01094-19.5 TABLE S2 Overview of RNase P/RNase mitochondrial RNA processing (MRP) subunits. Comparison of subunits of RNase P and RNase for MRP from S. cerevisiae (orange), H. sapiens (blue), and A. nidulans (green) (3, 4); the CandA-C1 protein is indicated in red letters. Download Table S2, DOCX file, 0.1 MB. Copyright © 2019 Köhler et al. 2019 Köhler et al. This content is distributed under the terms of the Creative Commons Attribution 4.0 International license.

BiFC microscopy and pulldown experiments showed that CandA-N/A-C single subunits interact with CandA-C1. The pulldown data also suggest that A. nidulans has a dimeric complex of CandA-N/A-C. Overexpression *candA-C1* in the absence of *candA-C* balanced the neddylation ratio of CulA and complemented the conidiation defect. These results underline the idea that CandA-C1 interacts with CandA-N. The two proteins together can partially take over functions of the CandA-N/A-C or CandA-N/A-C1/A-C complexes. The stoichiometry of the complexes needs further investigation. The deneddylation assay supports the idea that all three A. nidulans CandA proteins are required for CRL disassembly, are obligatory for CRLs that lack a substrate, and are therefore deneddylated by the CSN. Disassembled and deneddylated cullins can bind other adaptor-receptor complexes for new substrate ubiquitination cycles, allowing the ubiquitination of diverse substrates involved in different cellular pathways, like the A. nidulans carbon catabolite repression, as recently shown (35). The CandA proteins are essential for optimal CRL activity, as was also shown by a mathematical modeling investigation by Liu and coworkers (15, 16).

This work demonstrates that CandA-C1 nuclear import is independent of CandA-C. Pulldowns revealed that CandA-C1 interacts with the importin-α/β1 homologs KapA (AN2142) and KapB (AN0906), which have nuclear and perinuclear localization, respectively (36, 37). A. nidulans KapA transports the master regulator of secondary metabolism VeA in complex with VelB into the nucleus (38–40). CandA-C1’s nucleolar localization might be due to interaction with the nonessential KapJ that was reported to have a nucleocytoplasmic and nucleolar localization (37). CandA localized to mitochondria, indicating that it regulates the activity of CRLs that are connected to the outer mitochondrial membrane (41, 42). The deletion of *candA* genes caused fragmented mitochondria (Fig. S3G), similar to what was reported for a strain lacking the proteasome lid subunit SemA (43). The observed mitochondrial dysfunctions might depend on CandA being required for development and secondary metabolism, and on CandA-C1 as essential vegetative growth factor, which might provide additional RNase-associated functions. A connection between asexual development and secondary metabolism was also shown recently for the transcription factor SclB that activates the central regulatory pathway for conidiation (44). All three CandA subunits are obligatory for the multicellular development of sexual ascospores, supporting the idea that regulation of the CRL cycle is required for complex organisms. This corroborates with the embryonic lethality of *csn* or *cand* malfunction in higher eukaryotes (45–48). A. fumigatus CanA NTE is obligatory for growth, and the CanA complex is required to cope with low-temperature stress. It is known that the major stress resistance factor for spores in A. fumigatus is trehalose (49). Whether CanA mediates trehalose stability through the UPS to improve stress resistance needs further investigation. The spread of fungal pathogens is difficult to control (50). The discovered impact of CanA on growth and the fact that the CandA-C1 protein domain is not conserved in higher eukaryotes could be beneficial for drug design against *Aspergillus*-derived diseases, like aspergillosis.

CandA-C1 coordinates secondary metabolite genes other than CandA-N and CandA-C. The Δ*candA-C* and Δ*candA-N* mutant strains produced cichorine and an unknown metabolite. From the mass of *m/z* 210.0761 [M+H]^+^, the molecular formula C_10_H_11_NO_4_ was deduced. A literature search indicated that this substance is most probably emerimidine, which is related to cichorine but was not identified in A. nidulans so far. Emerimidine produced by Emericella variecolor CLB38 was shown to have antimicrobial activity against multidrug-resistant microorganisms like Bacillus subtilis and Staphylococcus aureus but also antifungal activity against Candida albicans and A. fumigatus (51). The thin-layer chromatogram of the extracts from asexually and sexually developed Δ*candA-C* and Δ*candA-N* mutants showed a blue spot at 366 nm at an *R_f_* of 0.43 (Fig. S3H), which is in accordance with the literature (51). The similarity between the UV-Vis spectra of cichorine and emerimidine, which fit very well with the literature, indicates that the two have the same core structure (51, 52). Cichorine is synthesized from a nonreducing polyketide synthase, CicF (AN6448) (52). This metabolite is a known phytotoxin, and compounds with a similar framework have been connected to antitumor and antimicrobial activities (52–54). Cichorine was also identified in a Δ*laeB* mutant strain that is impaired in sterigmatocystin synthesis (55, 56). Previous studies found orsellinic acid and derivatives in *candA, csn, veA*, and *velB* deletion strains and connected these compounds to the dark pigment secreted by those strains (22, 57, 58). These compounds were not identified in the present study under the growth and metabolite extraction conditions used. Austinol and dehydroaustinol from the *aus* gene cluster were nearly absent in *candA-C* and *candA-N* deletion strains, correlating with the reduced amount of conidia (59). Conidia were observed when *candA-C1* was missing, and austinol, dehydroaustinol, and emericellin, as well as xanthones from the *mdp* cluster, were isolated, which are all connected to asexual development and are present in the wild type (60). The metabolite asperthecin is known as the red pigment of cleistothecia (ascospores) and was found after 7 days in a sexually developed Δ*candA-C1* mutant strain, although no mature cleistothecia or ascospores were present, indicating that CandA-C1 supports the asperthecin synthesis but is impaired in the production of cleistothecia with mature ascospores (61).

This work demonstrates an evolutionarily changed CandA complex that comprises the newly identified 20-kDa CandA-C1 subunit in Aspergillus nidulans, which is part of the CanA C-terminal subunit in A. fumigatus. This additional subunit is required in both aspergilli for vegetative growth and asexual conidia formation (Fig. 9B and C). A. nidulans CandA proteins are required to fullfill the sexual life cycle and all together are a prerequisite for priming CRL assembly, conferring dynamic protein turnover. Therefore, a trimeric CandA complex is as possible as a dimer of CandA-N/A-C or CandA-N/A-C1, whereby CandA-C1 has a dual function in CRL regulation and putatively as an RNase P subunit. With all this information, *Aspergillus* CandA-C1 is an promising target to control fungal spread.

## MATERIALS AND METHODS

### Strains and media.

The oligonucleotides and plasmids used for strain design are described in the supplemental material (Text S1). Strains were cultivated in liquid or solid minimal medium (MM) (44). Modified minimal medium (32) was used for A. fumigatus spotting or Western hybridization experiments. Four thousand conidiospores were used for spot tests. A. nidulans plates were incubated at 37°C (if not indicated otherwise) in the light for asexual development, or plates were sealed with Parafilm and incubated in darkness for sexual development. Vegetative mycelium was obtained from liquid MM or modified MM cultures inoculated with 1 × 10^6^ to 2 × 10^6^ spores/ml at 37°C for 20 h with agitation. Conidia were quantified as described previously (44).

10.1128/mBio.01094-19.6 TEXT S1 Supplementary methods, like plasmid and strain design, as well as *in vitro* protein pulldown, in-gel digestion of proteins with trypsin, peptide analysis with LC-MS, Perseus workflow for protein analysis, and identification and thin-layer chromatography are described. Download Text S1, DOCX file, 0.1 MB. Copyright © 2019 Köhler et al. 2019 Köhler et al. This content is distributed under the terms of the Creative Commons Attribution 4.0 International license.

### Isolation of fungal genomic DNA and RNA and cDNA synthesis.

gDNA was extracted with DNA lysis buffer (200 mM Tris-HCl [pH 8.5], 250 mM NaCl, 25 mM EDTA, 0.5% [wt/vol] SDS) and 8 M potassium acetate solution from ground mycelia obtained from liquid overnight cultures. gDNA was precipitated with isopropanol and the pellets resolved in distilled water (dH_2_O). RNA was isolated according to the RNeasy plant minikit (Qiagen) protocol. cDNA was transcribed from 0.8 μg RNA using the QuantiTect reverse transcription kit (Qiagen).

### Gene expression measurements.

Transcription levels were analyzed by qRT-PCR with the primers from Table S3, using the equipment described previously (32). Expression levels were quantified relative to the housekeeping gene (*h2A*) with the ΔΔ*CT* method (62).

10.1128/mBio.01094-19.7 TABLE S3 Oligonucleotides designed and used in this study; p.c., personal communication. Download Table S3, DOCX file, 0.1 MB. Copyright © 2019 Köhler et al. 2019 Köhler et al. This content is distributed under the terms of the Creative Commons Attribution 4.0 International license.

### cDNA amplification assay.

PCRs were performed with 2 μl cDNA (∼2 μg) per reaction with the primers listed in Table S3 and Phusion high-fidelity DNA polymerase (Thermo Fisher Scientific), according to the manufacturer’s instructions. PCR fragments were analyzed by agarose gel electrophoresis.

### Protein extraction from A. nidulans and A. fumigatus.

Protein crude extracts, SDS-PAGE, and Western hybridization experiments were prepared as described before (25). See Text S1 for a detailed protocol of *in vitro* protein pulldown, in-gel digestion of proteins with trypsin, and peptide analysis with LC-MS. For this study, the primary antibodies anti-GFP (B-2, catalog no. sc-9996; Santa Cruz), anti-red fluorescent protein (anti-RFP; 5F8; Chromotek), anti-hemagglutinin (anti-HA, clone HA-7; Sigma-Aldrich), anti-CulA, anti-Nedd8 (both GeneScript), anti-ubiquitin 05-944 (Merck), or anti-tubulin antibody T0926 (Sigma-Aldrich) were used. Anti-goat mouse (115-035-003; Jackson Immunoresearch) or anti-goat rabbit (G21234; Invitrogen) served as secondary antibodies.

### Microscopy.

Strain morphology was analyzed using an SZX12 stereo microscope (Olympus) and Axiolab light microscope (Zeiss). Liquid MM in an 8-well microscopy chamber (Ibidi) was inoculated with 500 spores and incubated for 18 to 24 h at 37°C for fluorescence microscopy. The microscope setup was described previously (44, 63). Nuclei were visualized with ectopically integrated *rfp*::*h2A* or stained with 0.1% (vol/vol) 4′,6-diamidino-2-phenylindole (DAPI). Mitochondria were stained with 50 nM MitoTracker Red (Invitrogen).

### Secondary metabolite extraction.

Two agar plates were inoculated with 10^6^ spores per strain and incubated for 7 days under asexual or sexual development-inducing conditions. Two plugs were punched out per plate with a 50-ml Falcon tube. The plugs were homogenized with a 20-ml syringe and mixed with 8 ml H_2_O (LC-MS grade; Merck) and 8 ml ethyl acetate (LC-MS grade; Roth) at 220 rpm agitation at 20°C overnight. Samples were centrifuged at 2,500 rpm for 10 min at 4°C. The upper phase was collected and evaporated. The remaining metabolites were reconstituted in methanol (LC-MS grade; Fisher Scientific) for LC-MS analysis.

### LC-MS analysis of secondary metabolites.

A Q Exactive Focus Orbitrap mass spectrometer coupled with an UltiMate 3000 high-performance liquid chromatography (HPLC; Thermo Fisher Scientific) was used to examine the reconstituted metabolites. The HPLC column (Acclaim 120, C_18_, 5 μm, 120 Å, 4.6 by 100 mm; Thermo Fisher Scientific) was loaded with 5 μl extract per sample and a linear acetonitrile with 0.1% (vol/vol) formic acid in H_2_O with 0.1% (vol/vol) formic acid gradient (from 5% to 95% [vol/vol] acetonitrile with 0.1% formic acid in 20 min, with an additional 10 min with 95% [vol/vol] acetonitrile with 0.1% formic acid) at a flow rate of 0.8 ml/min at 30°C was applied. The measurements were conducted in a mass range of *m/z* 70 to 1,050 in positive mode. For tandem MS (MS^2^) spectra, a stepped collision energy of 20, 30, and 40 eV was applied. Data were analyzed with Xcalibur 4.1 (Thermo Fisher Scientific) and FreeStyle 1.4 (Thermo Fisher Scientific).

10.1128/mBio.01094-19.8 TABLE S4 Plasmids designed and used in this study. A. nidulans genes are labeled with An_geneX and A. fumigatus genes with Af_geneX. ^P^, promoter; ^T^, terminator; ^R^, resistance; PP, PreScission cleavage site; L, linker; *bla*, ampicillin resistance gene; *nat*-RM, recyclable *nat* resistance cassette from pME4304 or pME4696; *phleo-*RM, recyclable *phleo* resistance cassette from pME4305; *ptrA-*RM, recyclable *ptrA* resistance cassette from pSK485 or pCHS314; p.c., personal communication. Download Table S4, DOCX file, 0.1 MB. Copyright © 2019 Köhler et al. 2019 Köhler et al. This content is distributed under the terms of the Creative Commons Attribution 4.0 International license.

10.1128/mBio.01094-19.9 TABLE S5 A. fumigatus and A. nidulans strains used in this study. ^P^, promoter; ^T^, terminator; PP, PreScission cleavage site; L, linker; *nat*^R^, nonrecyclable *nat* resistance; *phleo*^R^, nonrecyclable *phleo* resistance. Download Table S5, DOCX file, 0.1 MB. Copyright © 2019 Köhler et al. 2019 Köhler et al. This content is distributed under the terms of the Creative Commons Attribution 4.0 International license.
