# Supplementary figures and images for: Integration of Fungus-Specific CandA-C1 into a Trimeric CandA Complex Allowed Splitting of the Gene for the Conserved Receptor Exchange Factor of CullinA E3 Ubiquitin Ligases in Aspergilli
Source: mBio. 2019 Jun 18;10(3):e01094-19. doi: 10.1128/mBio.01094-19 (PMC6581859; doi:10.1128/mBio.01094-19)

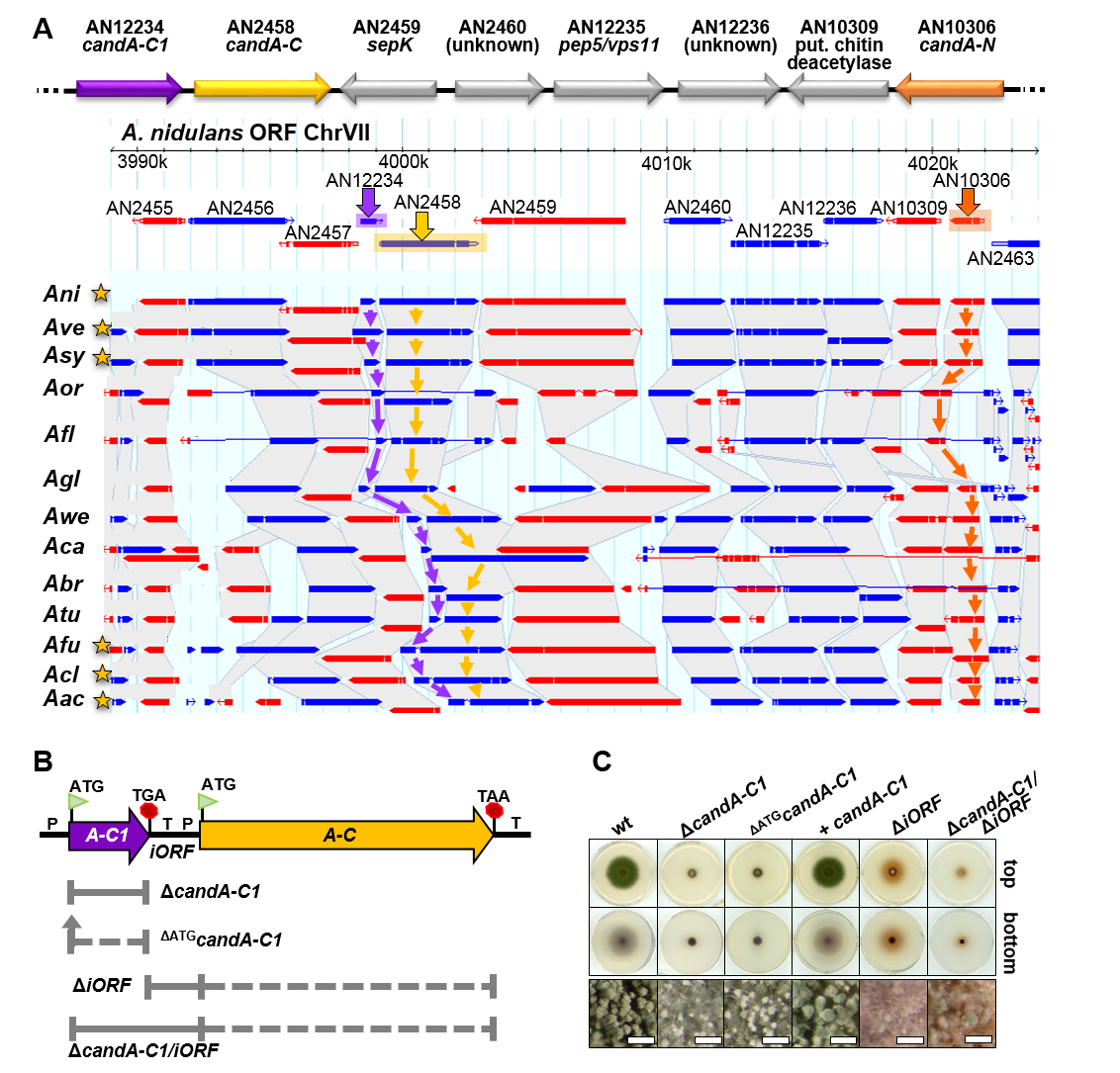

Supplement: FIG S1 [file mBio.01094-19-sf001.tif]

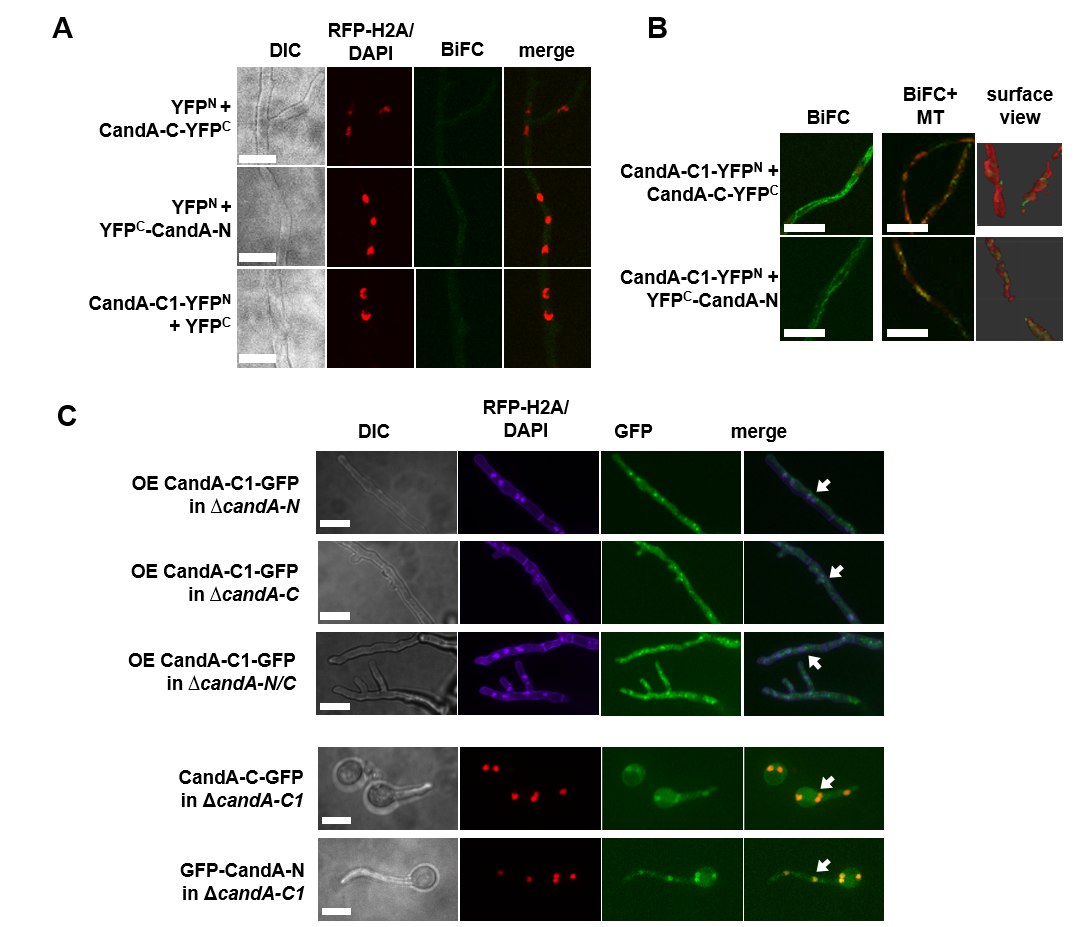

Supplement: FIG S2 [file mBio.01094-19-sf002.tif]

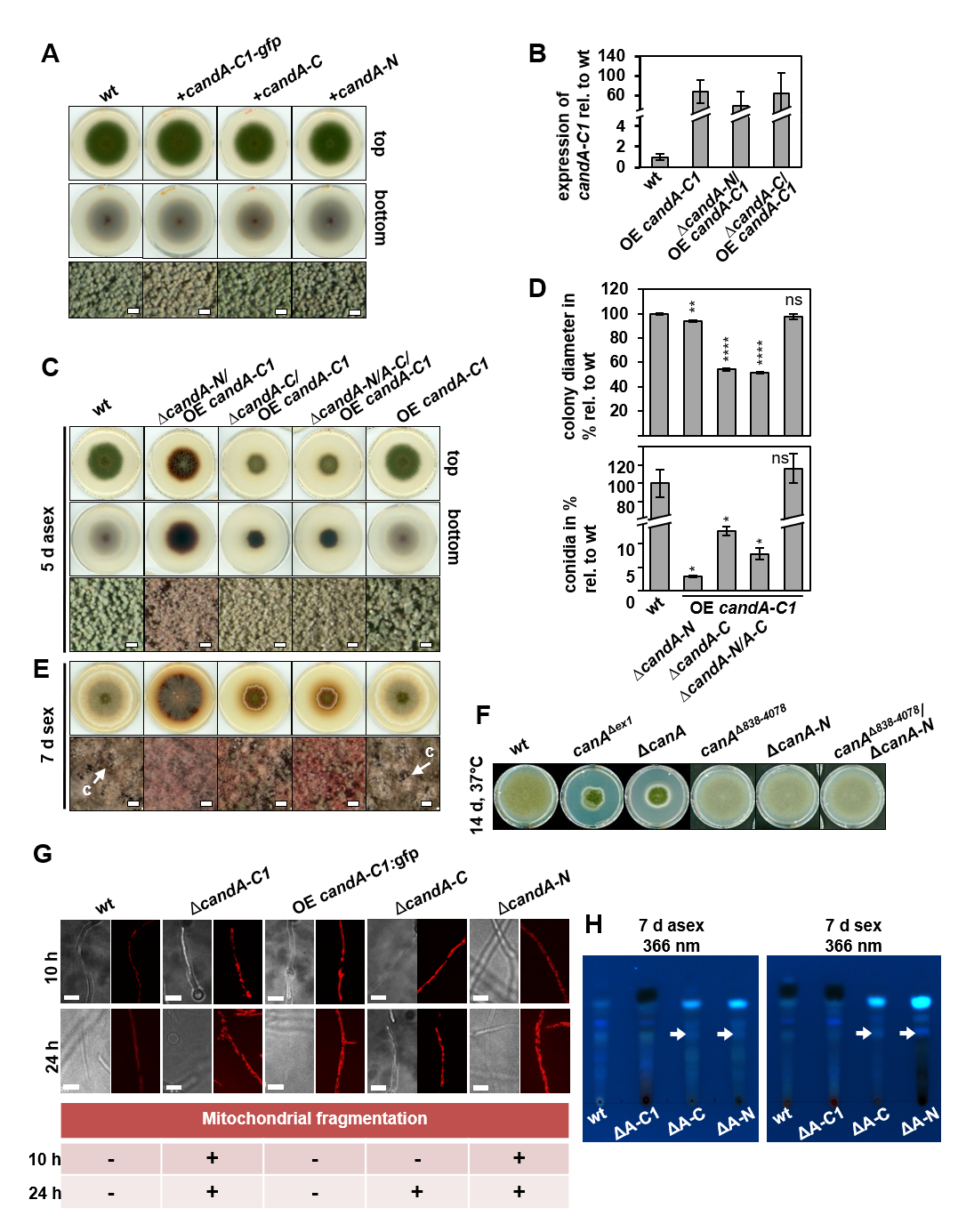

Supplement: FIG S3 [file mBio.01094-19-sf003.tif]
