# Supplementary material for: Integration of Fungus-Specific CandA-C1 into a Trimeric CandA Complex Allowed Splitting of the Gene for the Conserved Receptor Exchange Factor of CullinA E3 Ubiquitin Ligases in Aspergilli
Source: mBio. 2019 Jun 18;10(3):e01094-19. doi: 10.1128/mBio.01094-19 (PMC6581859; doi:10.1128/mBio.01094-19)
Supplement: TABLE S1 [file mBio.01094-19-st001.docx]

**TABLE S1** Identified proteins in *A. nidulans* CandA-N, CandA-C and CandA-C1 pull-downs. Detailed information about identified proteins from the heat map in Fig. 4A as result of filtering (Fig S3).

| Systematic name | Standard name | Description |
| --- | --- | --- |
| Nucleus | | |
| AN10306 | CandA-N | Cullin-associated NEDD8-dissociated protein 1, N-terminal part |
| AN2458 | CandA-C | Cullin-associated NEDD8-dissociated protein 1, C-terminal part |
| AN1019 | CulA | SCF ubiquitin ligase complex subunit CulA |
| AN2142 | KapA | Importin subunit alpha |
| AN0906 | KapB | Importin subunit beta1 |
| AN2120 | KapJ | Importin subunit beta-4 |
| AN7894 | - | Uncharacterized protein, alpha-beta barrel and YCII domain predict enzymatic function |
| AN7299 | Arx1 | Uncharacterized protein; curved DNA-binding protein, aminopeptidase activity, nuclear export |
| AN2343 | HbnA | Uncharacterized protein; nitroreductase family protein |
| AN8899 | - | Putative 1-aminocyclopropane-1-carboxylate deaminase |
| AN3031 | ThrC | Threonine synthase |
| mitochondrion | | |
| AN10745 | ShmA | Serine hydroxy methyltransferase |
| AN8273 | QcrB | Ubiquinol-cytochrome c reductase complex core protein 2 |
| AN7893 | EncD | Uncharacterized protein; oxidoreductase with Fe ligand |
| AN8770 | ArgEF | Acetylglutamate kinase |
| AN7000 | LscB | Succinyl-CoA synthetase beta subunit, |
| AN10901 | GcvB | Uncharacterized protein; glycine cleavage system P protein |
| AN2999 | IdpA | Isocitrate dehydrogenase |
| AN1923 | AltA | Alanine transaminase |
| AN10296 | FrdA | Putative FAD dependent oxidoreductase |
| AN9403 | PdhC | Pyruvate dehydrogenase E1 component, beta subunit |
| AN0252 | AtpC | ATP synthase subunit gamma |
| AN8953 | AgdB | Uncharacterized protein; glycosidase and hydrolase activity |
| AN6246 | CycA | Cytochrome c |
| AN7897 | DbaB | FAD binding domain protein of the *dba* secondary metabolite gene cluster |
| AN7895 | CipB | Zinc-binding alcohol dehydrogenase domain-containing protein, concanamycin-induced protein B |
| AN7710 | - | Uncharacterized protein; HAD-superfamily hydrolase |
| AN7169 | FhbA | Uncharacterized protein; expressed flavohemoprotein |
| AN5162 | PdhA | Pyruvate dehydrogenase E1 component subunit alpha |
| AN4430 | - | Uncharacterized protein; acetolactate synthase activity, role in branched-chain amino acid biosynthetic process |
| AN3344 | Ngn27 | Uncharacterized protein; acetyltransferase activity |
| cytosol | | |
| AN8224 | GusA | Glutamyl-tRNA synthetase |
| AN6126 | AccA | Uncharacterized protein; acetyl-CoA carboxylase activity |
| AN2286 | AlcC | Alcohol dehydrogenase 3 (ADH III) |
| AN9339 | CatB | Catalase B |
| AN4888 | PdcA | Pyruvate decarboxylase |
| AN6231 | TrpB | Bifunctional tryptophan synthase TRPB |
| AN5886 | LuA | 3-isopropylmalate dehydratase (Alpha-IPM isomerase) |
| AN5571 | KgdA | Uncharacterized protein; Oxoglutarate dehydrogenase (Succinyl-transferring) |
| AN3223 | PfkA | 6-phosphofructokinase |
| AN2243 | CpaA | Carbamoyl-phosphate synthase arginine-specific small chain (CPS-A) |
| AN2743 | eIF-3a | Eukaryotic translation initiation factor 3 subunit A (eIF3a) |
| AN0359 | eIF-3b | Putative eIF3b subunit of translation initiation factor 3 (eIF3), required for conidial germination |
| AN7105 | eIF-3c | Eukaryotic translation initiation factor 3 subunit C (eIF3c) |
| AN2907 | eIF-3e | Eukaryotic translation initiation factor 3 subunit E (eIF3e) |
| AN10182 | eIF-3f | Eukaryotic translation initiation factor 3 subunit F (eIF3f) |
| AN5954 | eIF-3l | Eukaryotic translation initiation factor 3 subunit L (eIF3l) |
| AN4259 | eIF-3m | Eukaryotic translation initiation factor 3 subunit M (eIF3m) |
| AN4908 | Tif31 | Clustered mitochondria protein homolog |
| AN4916 | RpsG | 40S ribosomal protein S7 |
| AN3702 | Cdc60 | Uncharacterized protein; Leucyl-tRNA synthetase |
| AN2149 | Cct1α | T-complex protein 1 subunit alpha, protein folding |
| AN0381 | Cct1β | T-complex protein 1, beta subunit, protein folding |
| AN1904 | Cct1Ɛ | T-complex protein 1, epsilon subunit, protein folding |
| AN5713 | Cct1η | T-complex protein 1, eta subunit, protein folding |
| AN1851 | Cct1θ | T-complex protein 1, theta subunit, protein folding |
| AN6688 | SepB | Septin B |
| AN4463 | ChcA | Clathrin heavy chain |
| AN1126 | ArfA | ADP-ribosylation factor |
| AN6004 | VipA | Actin cytoskeleton protein (VIP1) |
| AN0285 | PglA | Uncharacterized protein; 6-phosphogluconolactonase |
| AN7193 | LarA | Uncharacterized protein; D-xylose reductases |
| extracellular | | |
| AN6048 | AatB | Uncharacterized protein; Aspartate transaminase |
| membrane | | |
| AN7902 | DbaH | Uncharacterized protein; FAD binding monooxygenase of *dba* secondary metabolite gene cluster |
