# Supplementary material for: Integration of Fungus-Specific CandA-C1 into a Trimeric CandA Complex Allowed Splitting of the Gene for the Conserved Receptor Exchange Factor of CullinA E3 Ubiquitin Ligases in Aspergilli
Source: mBio. 2019 Jun 18;10(3):e01094-19. doi: 10.1128/mBio.01094-19 (PMC6581859; doi:10.1128/mBio.01094-19)
Supplement: DATA SET S1 [file mBio.01094-19-sd001.pdf]

Data Set 1. EIC (extracted ion chromatogram), MS2 and UV/VIS spectra of identified secondary metabolites from asexual and sexual development of *A.nidulans* wild type (wt) and *candA* deletion strains.

Asexual development I) Austinol (1)

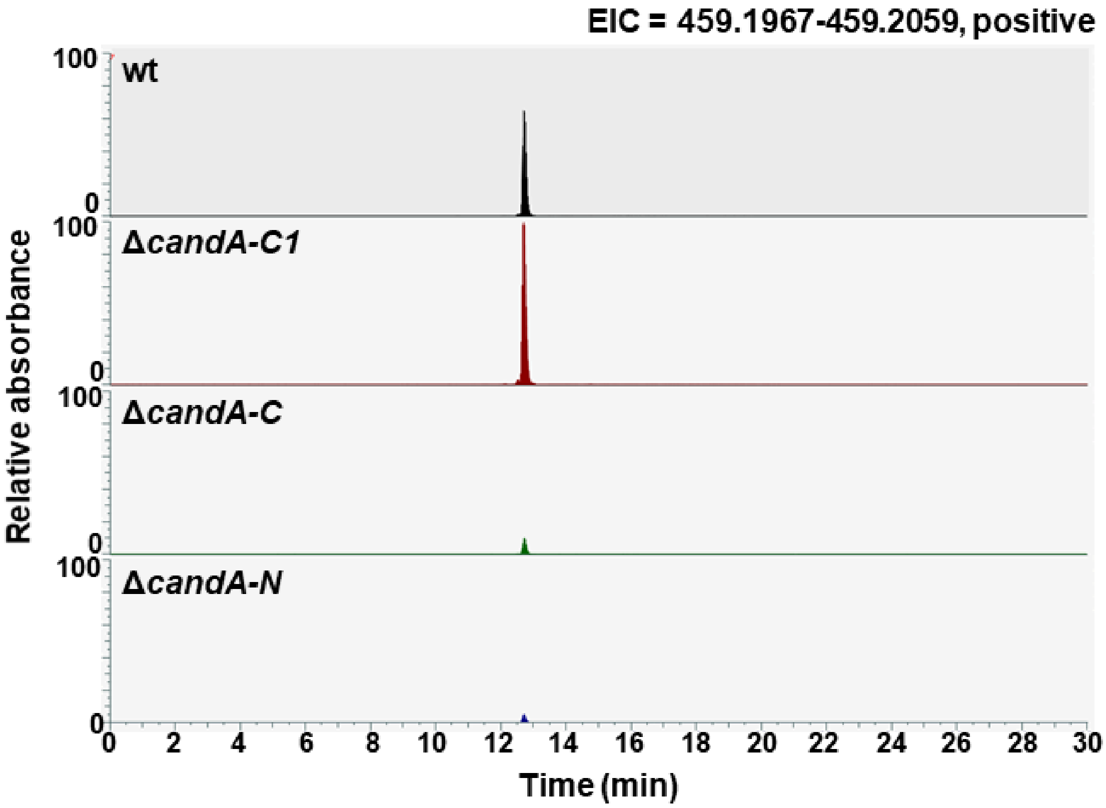

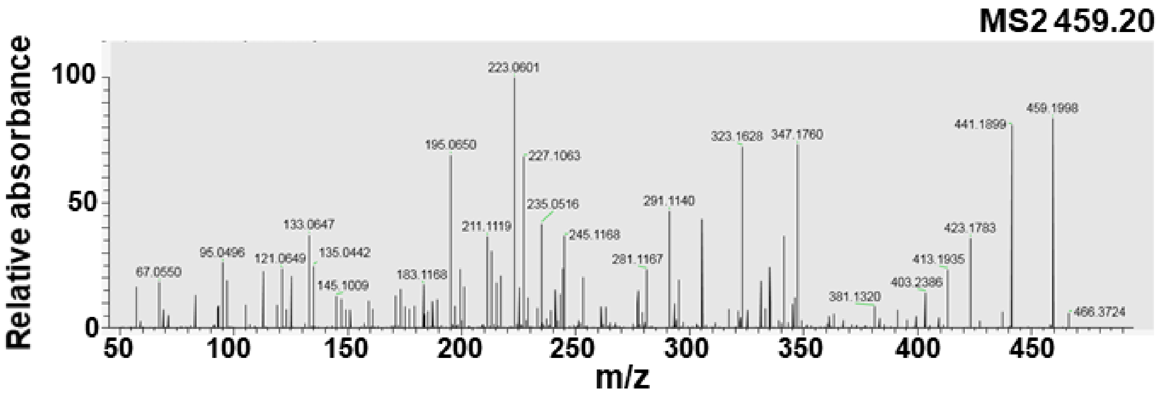

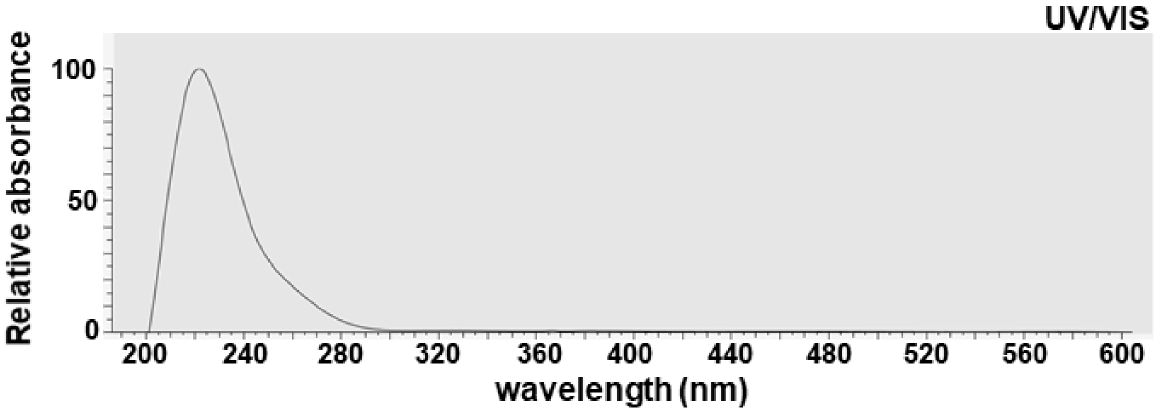

Asexual development II) Dehydroaustinol (1)

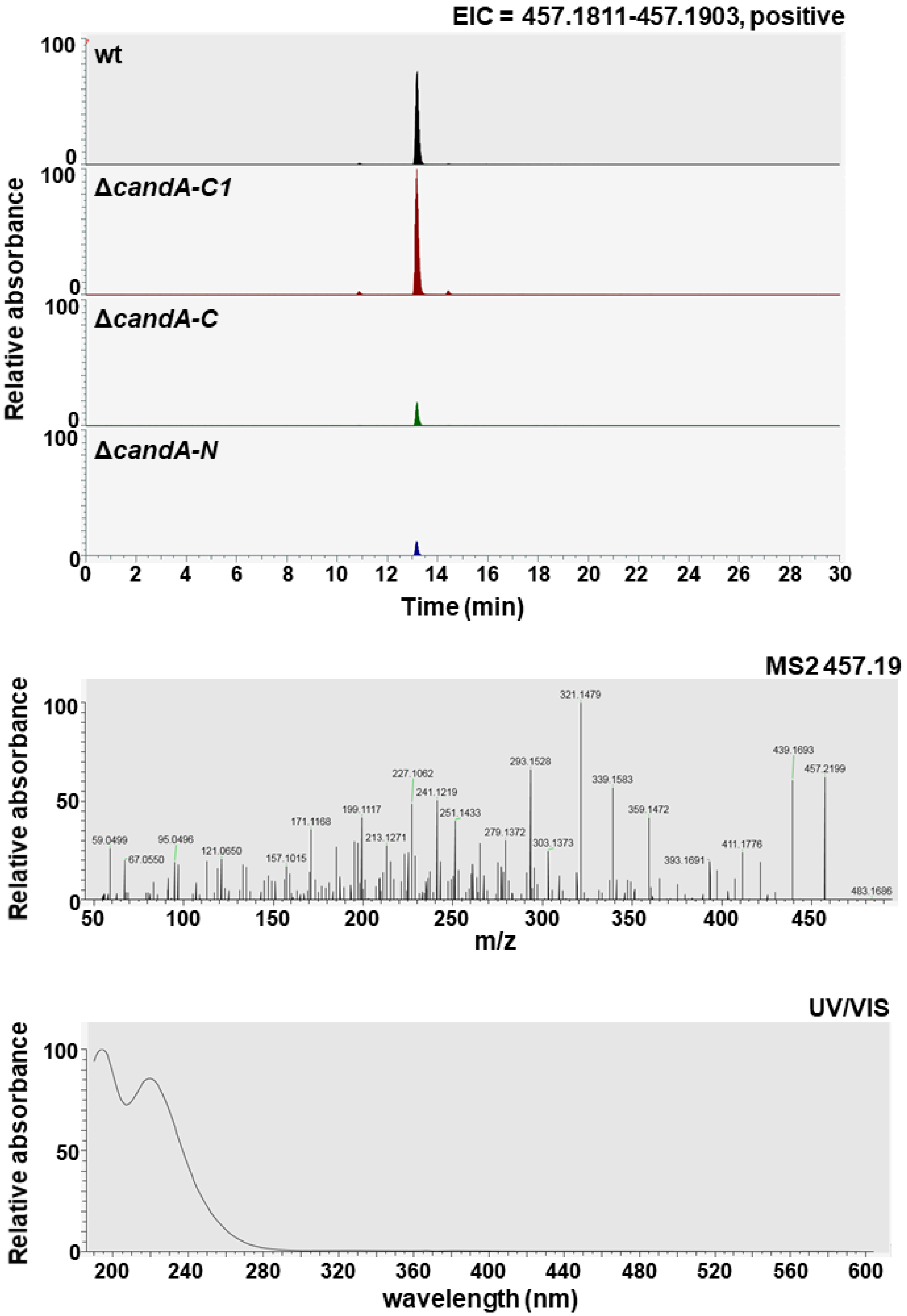

Asexual development III) Asperthecin (2)

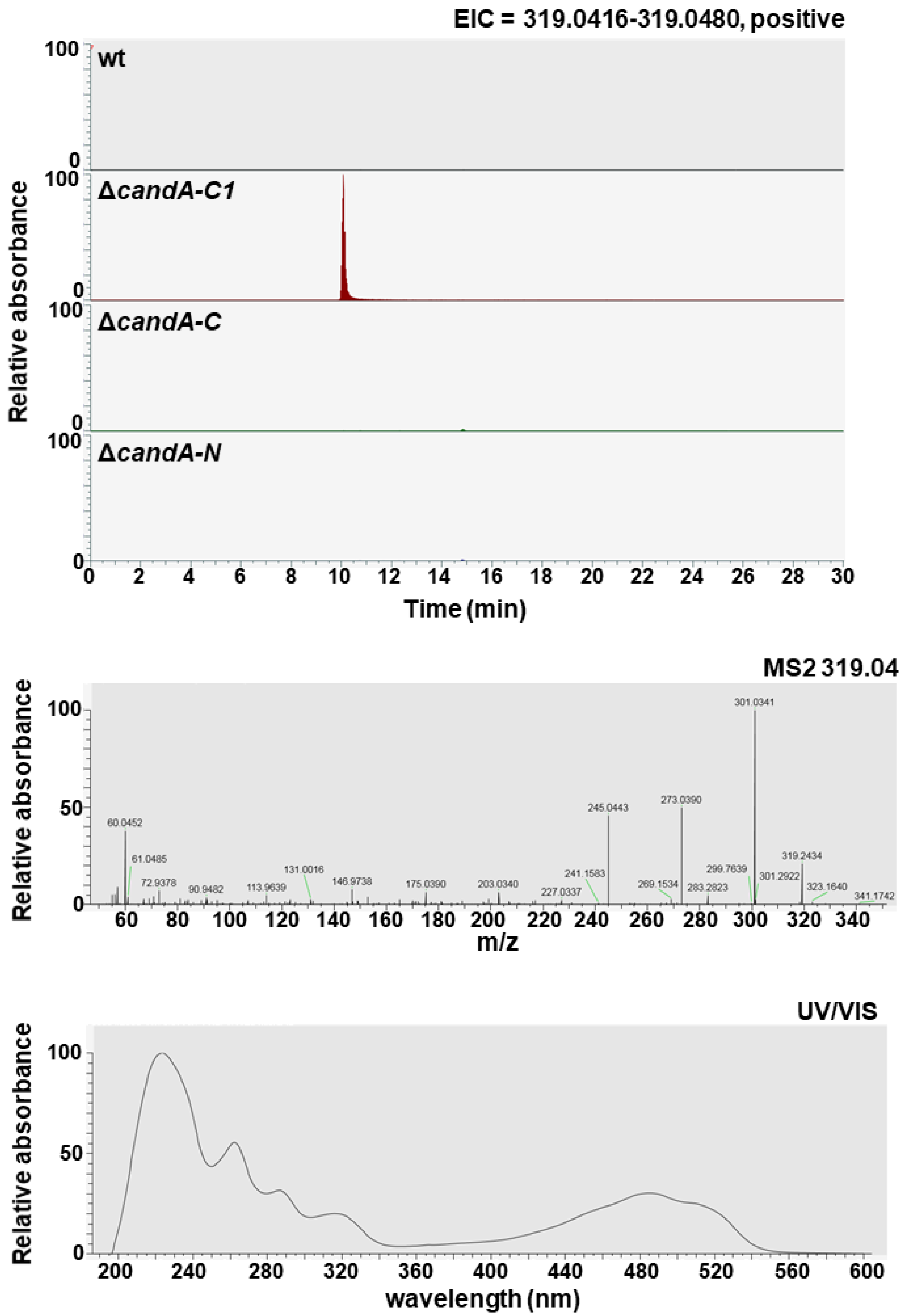

Asexual development IV) Emericellin (2)

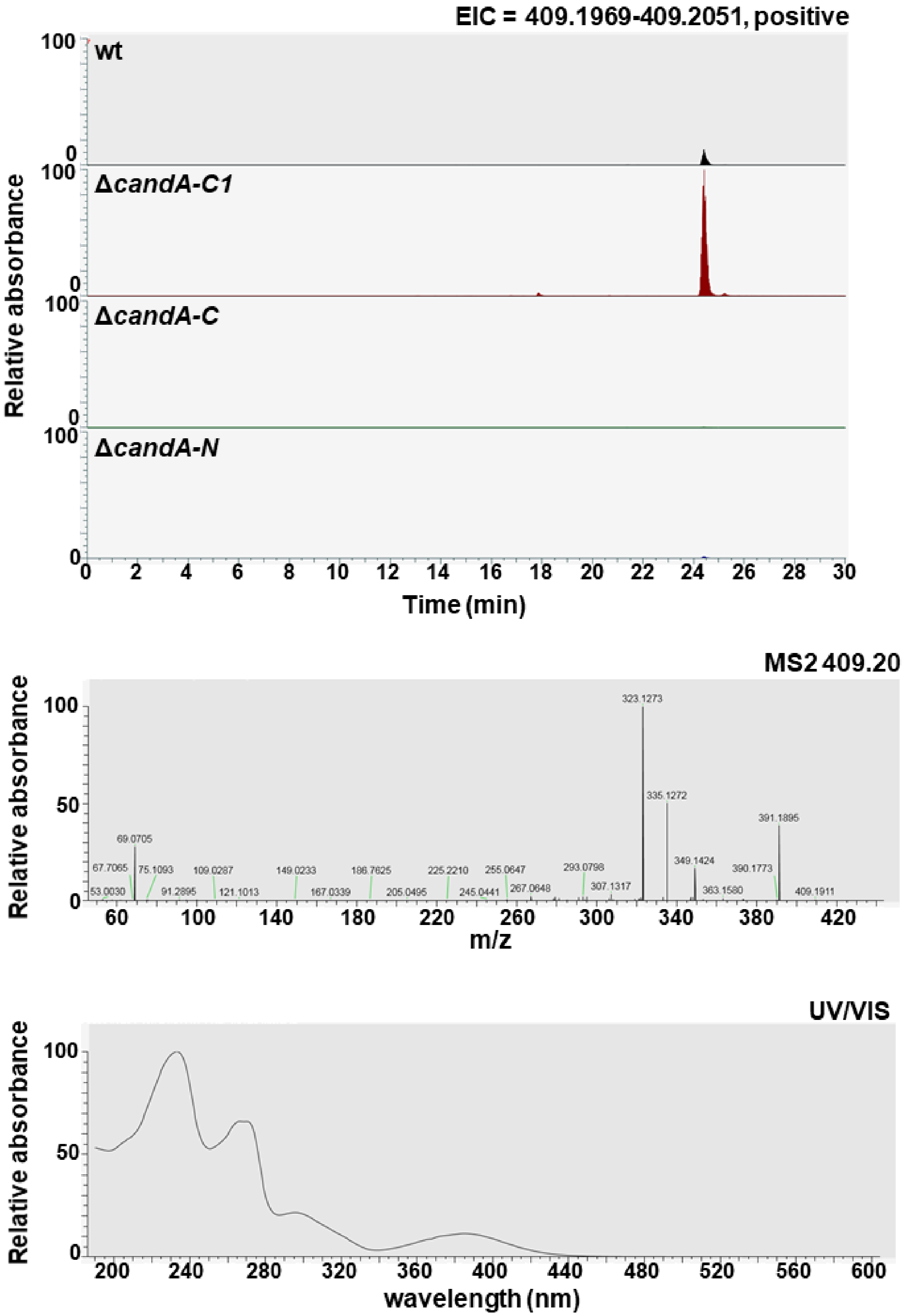

Asexual development V) Shamixanthone/ VI) Epishamixanthone (2)

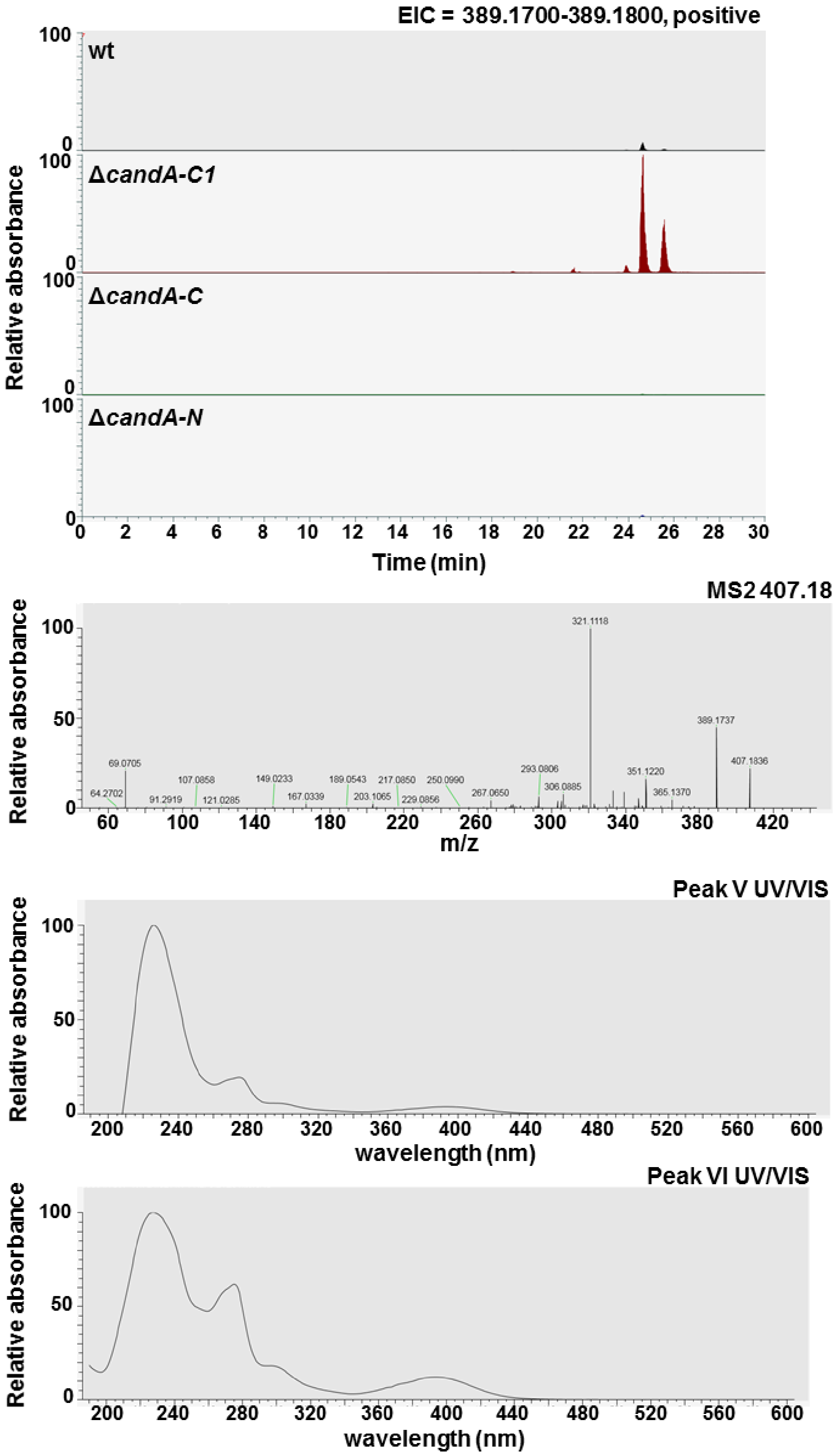

Asexual development VII) Cichorine (3)

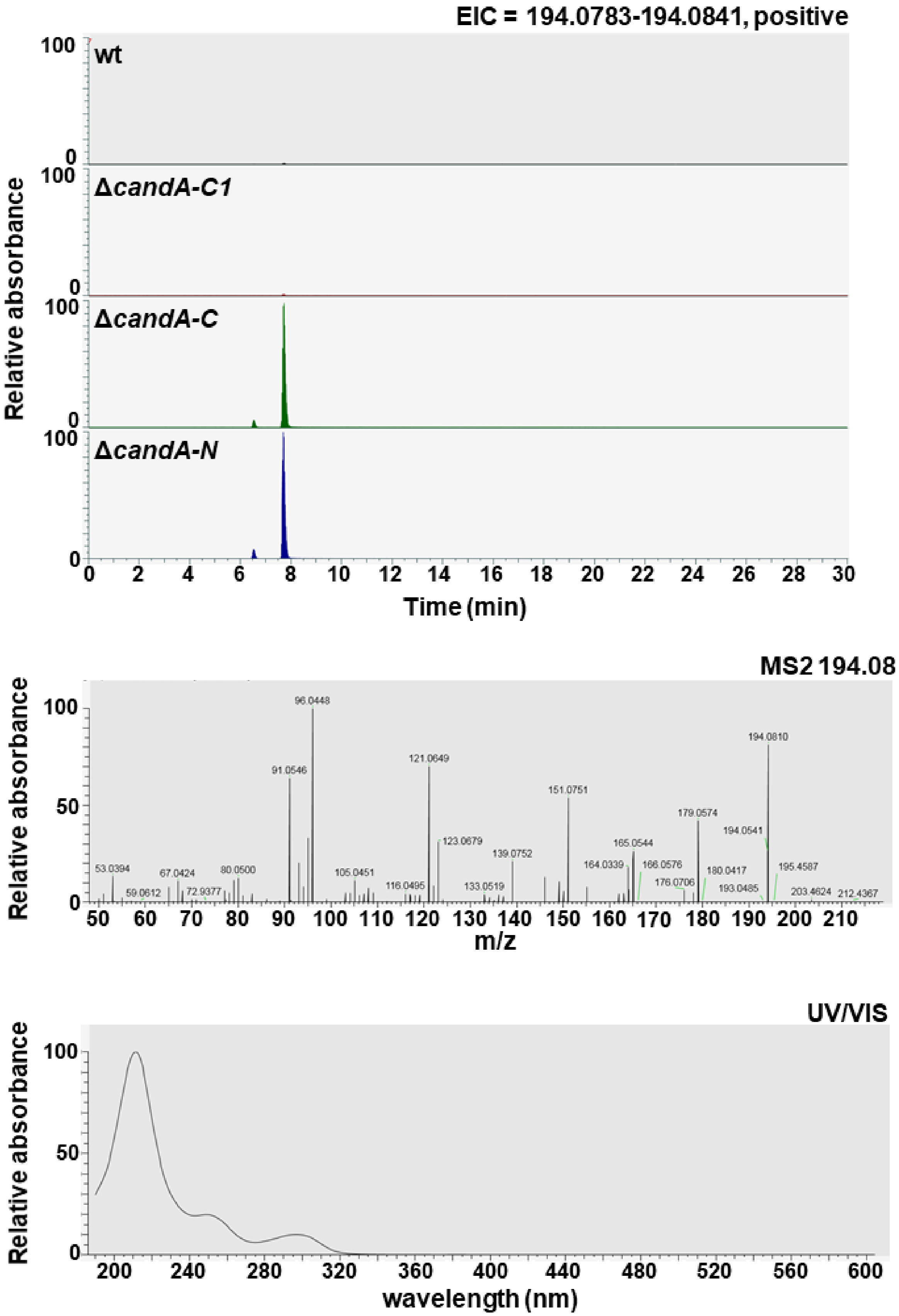

Asexual development VIII) Emerimidine A/B (4)

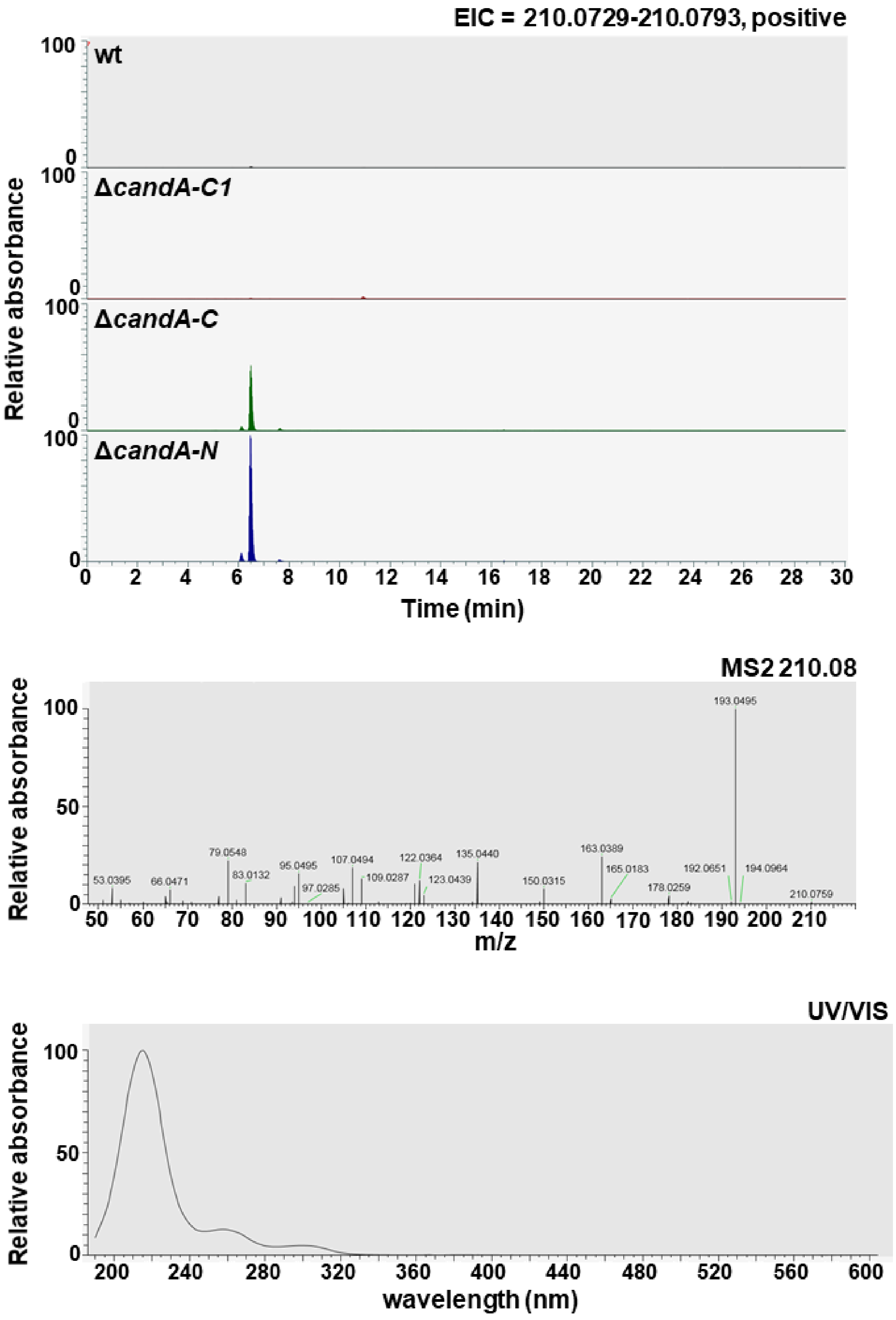

Sexual development III) Asperthecin (2)

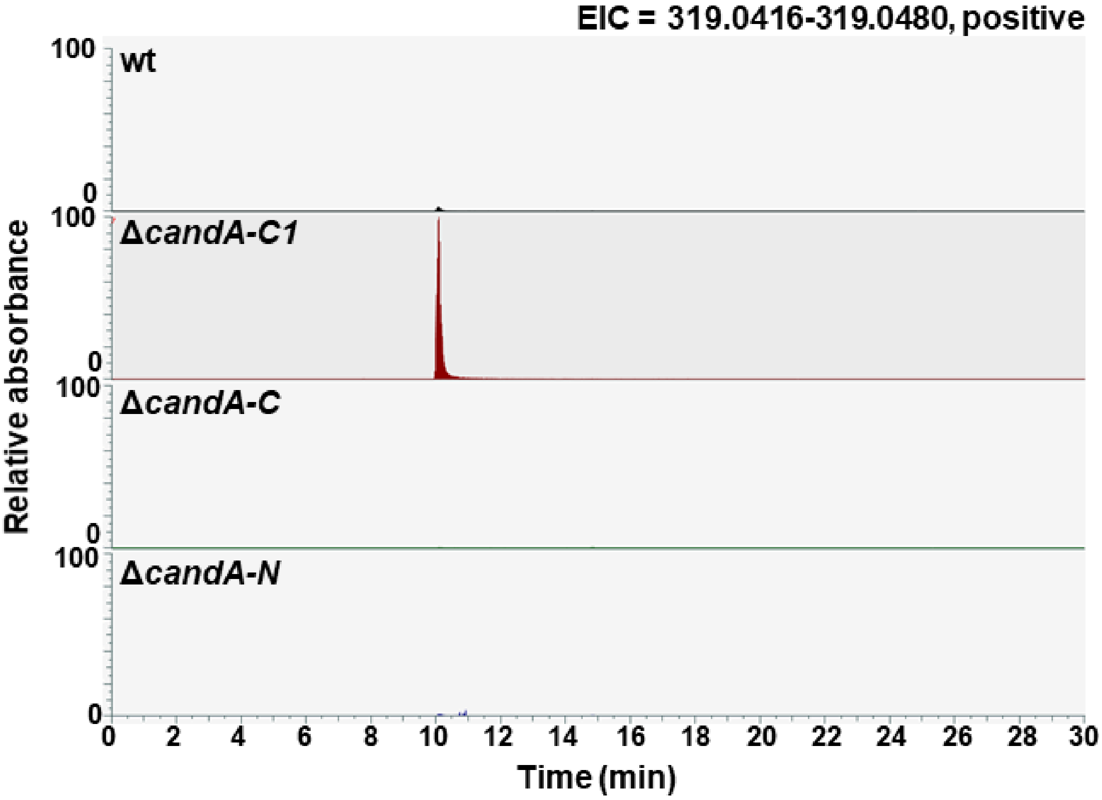

Sexual development IV) Emericellin (2)

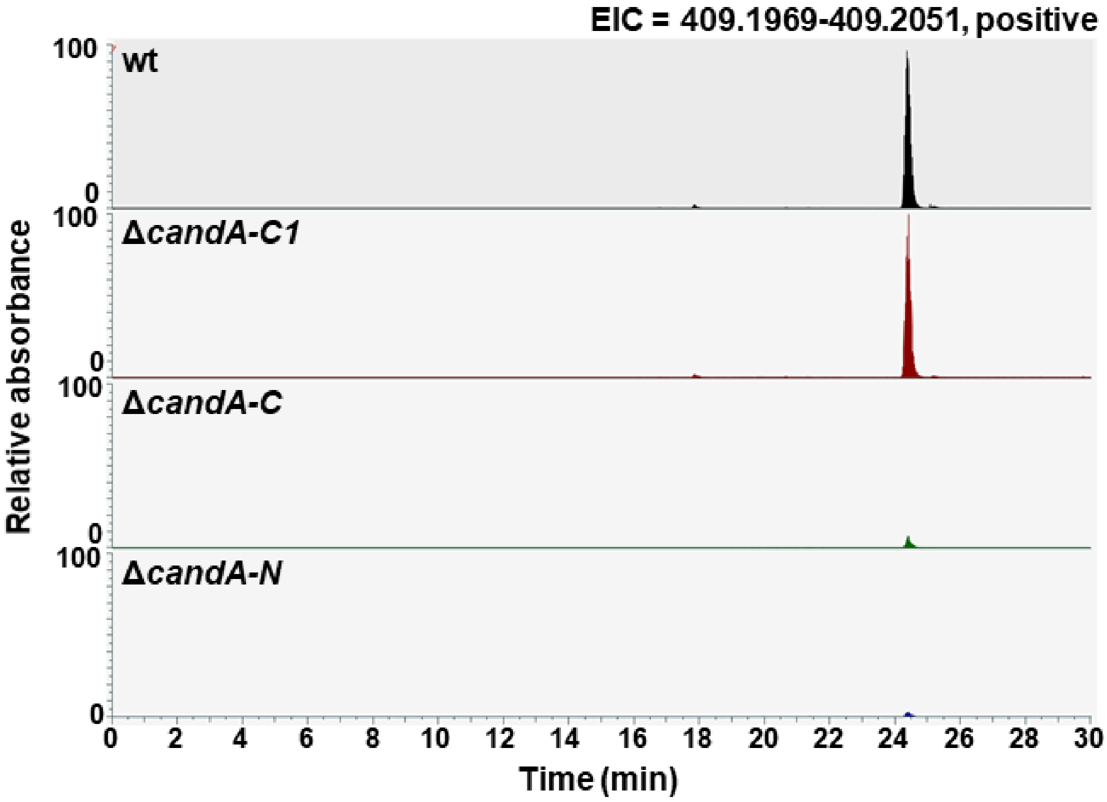

Sexual development V) Shamixanthone/ VI) Epishamixanthone (2)

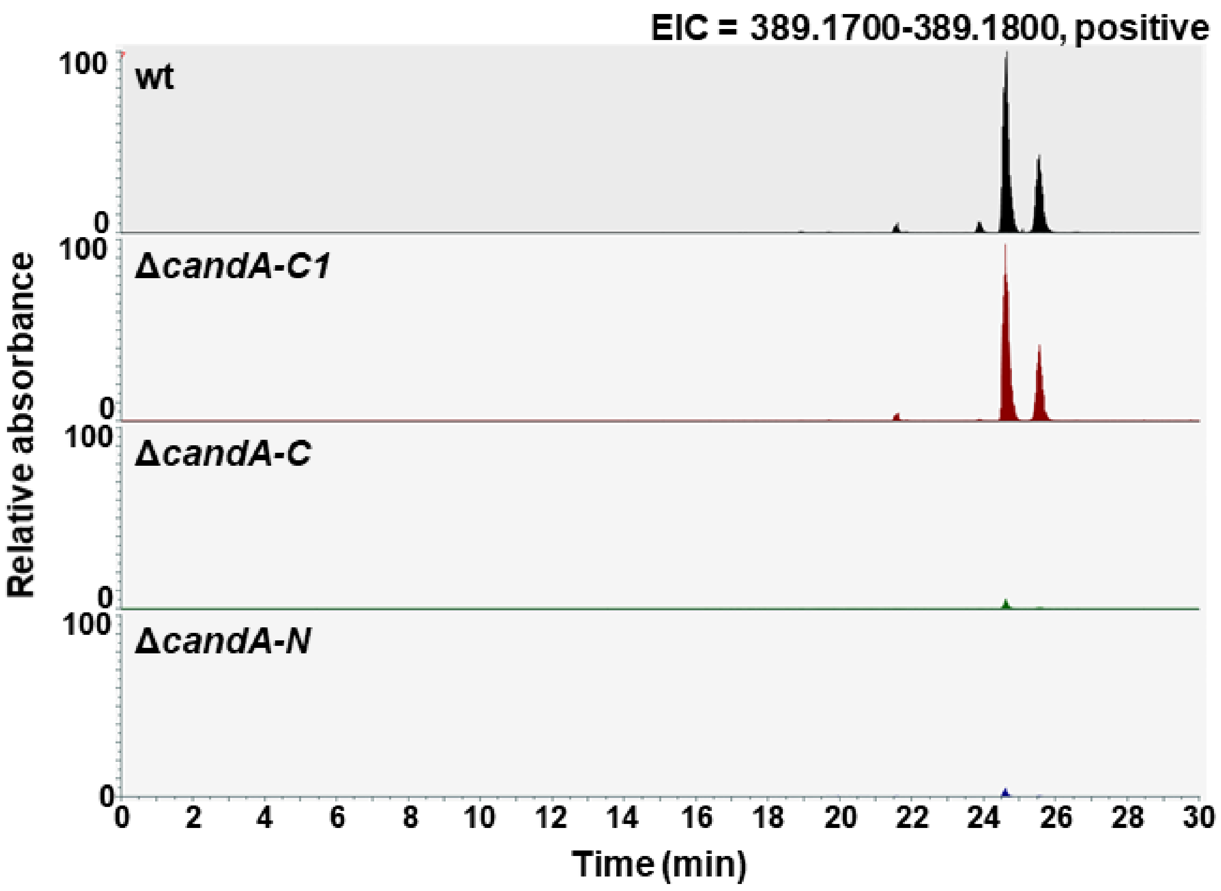

Sexual development VII) Cichorine (3)

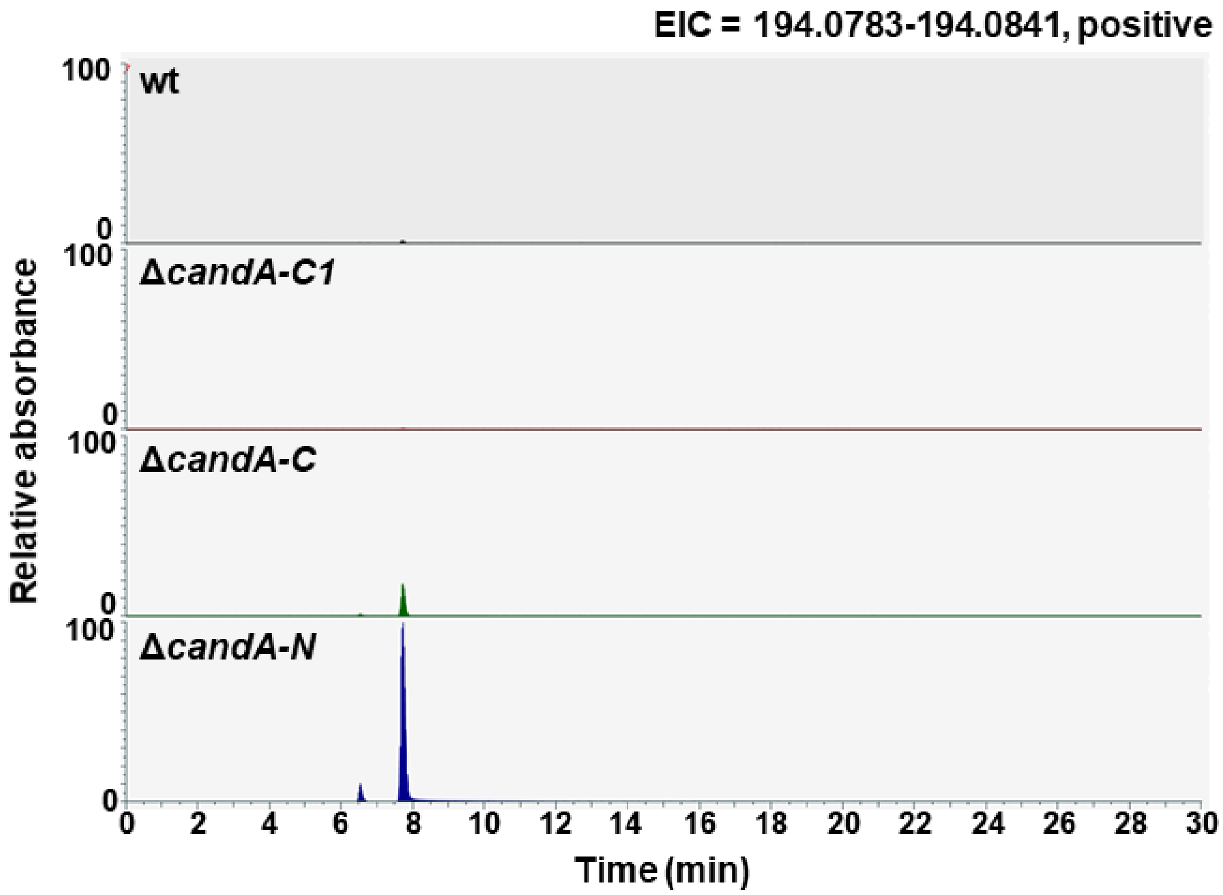

Sexual development VIII) Emerimidine (4)

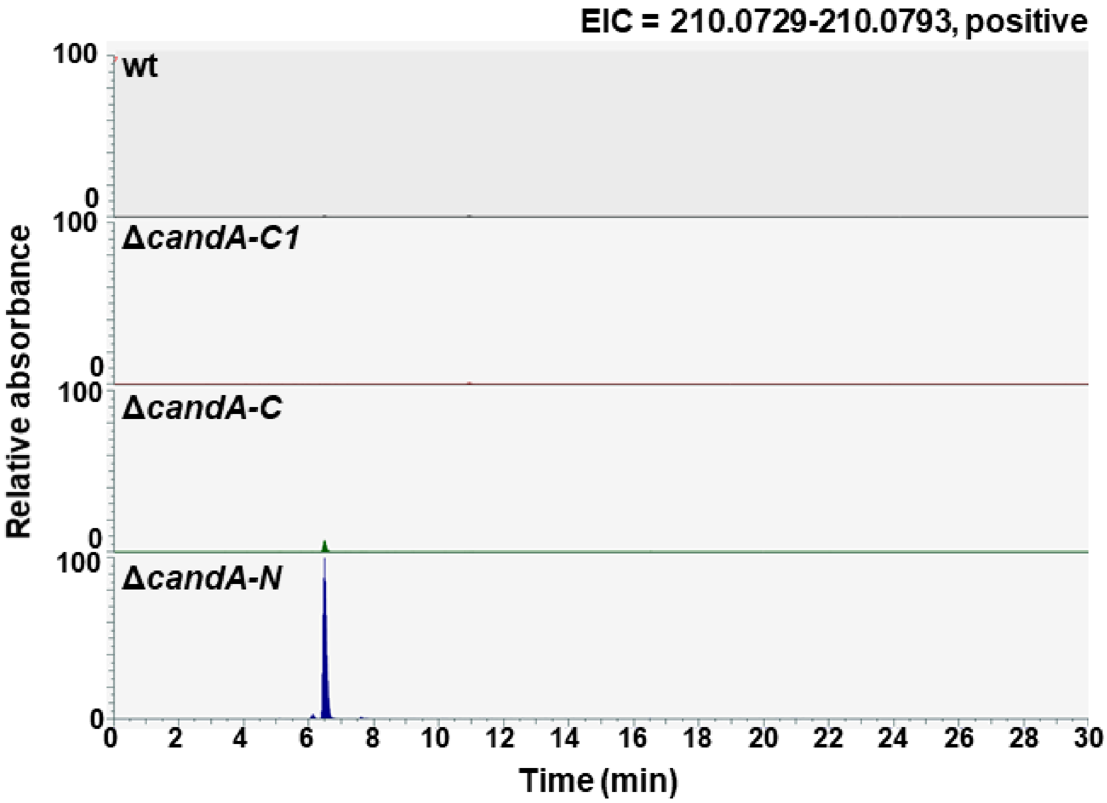

References

1. Albright JC, Henke MT, Soukup AA, McClure RA, Thomson RJ, Keller NP, Kelleher NL. 2015. Large-Scale Metabolomics Reveals a Complex Response of *Aspergillus nidulans* to Epigenetic Perturbation. ACS Chem Biol 10:1535–1541.

2. Bayram Ö, Feussner K, Dumkow M, Herrfurth C, Feussner I, Braus GH. 2016. Changes of global gene expression and secondary metabolite accumulation during light-dependent *Aspergillus nidulans* development. Fungal Genet Biol 87:30–53.

3. Sanchez JF, Entwistle R, Corcoran D, Oakley BR, Wang CCC. 2012. Identification and

molecular genetic analysis of the cichorine gene cluster in *Aspergillus nidulans*. Medchemcomm 3:997–1002.

4. Zhang G, Sun S, Zhu T, Lin Z, Gu J, Li D, Gu Q. 2011. Antiviral isoindolone derivatives from an endophytic fungus *Emericella* sp. associated with *Aegiceras corniculatum*. Phytochemistry 72:1436–1442.
