# Supplementary material for: Integration of Fungus-Specific CandA-C1 into a Trimeric CandA Complex Allowed Splitting of the Gene for the Conserved Receptor Exchange Factor of CullinA E3 Ubiquitin Ligases in Aspergilli
Source: mBio. 2019 Jun 18;10(3):e01094-19. doi: 10.1128/mBio.01094-19 (PMC6581859; doi:10.1128/mBio.01094-19)
Supplement: TABLE S2 [file mBio.01094-19-st002.docx]

| subunit type | *S. cerevisiae* | | *H. sapiens* | | *A. nidulans* | | |
| --- | --- | --- | --- | --- | --- | --- | --- |
|  | **RNase P** | **RNase MRP** | **RNase P** | **RNase MRP** | **RNase P** | **RNase MRP** | **size** |
| RNA | Rpr1/  Rnh1 | - | H1 | - | AN5188 ? | - | 1389 bp |
| RNA | - | Rpm1 | - | 7-2 | - | AN20050 Mpr1 | 232 bp |
| Protein | Pop1 | Pop1 | Pop1 | Pop1 | AN8691 | AN8691 | 101.9 kDa |
| Protein | Pop3 | Pop3 | - | - | - |  | - |
| Protein | Pop4 | Pop4 | Rpp29 | Rpp29 | AN0189 | AN0189 | 31.1 kDa |
| Protein | Pop5 | Pop5 | Rpp14 | Rpp14 | AN2437 | AN2437 | 23.6 kDa |
| Protein | Pop6 | Pop6 | - | - | - | - | - |
| Protein | Pop7 | Pop7 | Rpp20 | Rpp20 | AN4631 | AN4631 | 31.1 kDa |
| Protein | Pop8 | Pop8 | - | - | - | - | - |
| Protein | Rpp1 | Rpp1 | Rpp30 | Rpp30 | AN1486 | ? | 33.3 kDa |
| Protein | Rpr2 | Rpr2 | Rpp21 | Rpp21 | AN12234  CandA-C1 | ? | 19.6 kDa |
| Protein | - | - | Rpp25 | Rpp25 | - | - | - |
| Protein | - | - | Rpp14 | Rpp14 | - | - | - |
| Protein | - | - | Rpp38 | Rpp38 | - | - | - |
| Protein | - | - | Rpp40 | Rpp40 | AN3652 | ? | 43.0 kDa |

**TABLE S2** Overview of RNase P/RNase MRP subunits. Comparison of subunits of RNase P and RNase for mitochondrial RNA processing (MRP) from *S. cerevisiae* (organge), *H. sapiens* (blue) and *A. nidulans* (green) (3, 4); the CandA-C1 protein is indicated in red letters.
