# Supplementary material for: Integration of Fungus-Specific CandA-C1 into a Trimeric CandA Complex Allowed Splitting of the Gene for the Conserved Receptor Exchange Factor of CullinA E3 Ubiquitin Ligases in Aspergilli
Source: mBio. 2019 Jun 18;10(3):e01094-19. doi: 10.1128/mBio.01094-19 (PMC6581859; doi:10.1128/mBio.01094-19)
Supplement: TEXT S1 [file mBio.01094-19-s0001.docx]

**TEXT S1**

***A. fumigatus* and *A. nidulans* strain design.** Gene targeting by homologous recombination was mostly performed with recyclable marker (RM) cassettes (5). Oligonucleotides are indicated in Table S3, Plasmids in Table S4 and strains in Table S5.

**Plasmid and strain construction of *A. fumigatus canA*^Δ^*^exon1^* deletion.** The 5’ flanking region was amplified using oAMK247/248 (2147 bp) and the 3’ flanking region of *canA^exon1^* using oAMK249/250 (2429 bp). Both fragments were ligated with the *ptrA-RM* into linear pUC19 resulting in pME4672. AfS35 was transformed with the *Pme*I excised cassette (9814 bp) resulting in AfGB140 after recycling the marker.

**Plasmid and strain construction of *A. fumigatus* Δ*canA.*** For full gene deletion of *canA* the 3’ flanking region was amplified with oAMK267/268 (781 bp) and ligated to *Pml*I linearized pCHS314. The plasmid was linearized with *Swa*I and ligated with the 5’ flanking region of *canA,* which was amplified with oAMK273/274 (2147 bp). The resulting plasmid pME4677 was cleaved with *Pme*I and the cassette (8168 bp) was used for transformation with AfS35 resulting in AfGB141 after marker recycling.

**Plasmid and strain construction of *A. fumigatus canA*^Δ^*^exon1^* complementation with *A. nidulans* *candA-C1.*** For complementing *canA***^Δ^*^exon1^*** with *A. nidulans candA-C1* the 5’ flanking region of *canA* was amplified with oAMK247/262 (2147 bp) and fused to *candA-C1*, amplified with oAMK252/139 (576 bp). The resulting fragment (2693 bp) was ligated with 3’ flanking region (oAMK249/250) and *ptrA-RM* to pUC19 resulting in pME4673. AfS35 was transformed with the *Pme*I excised cassette (10360 bp) resulting in AfGB142 after recycling the marker.

**Plasmid and strain construction of *A. fumigatus* CanA-GFP fusion construct.** The 5’ flanking region of *canA* was amplified together with *canA* using oAMK269/270 (4595 bp). The PreScission protease cleavage site (PP; sequence: LEVLFQGP) and a linker (L; sequence: GGSGG) were amplified from pME4652 using oAMK82/271 (773 bp) and then both fragments were ligated to *Swa*I linearized plasmid containing the 3’ flanking region of *canA.* The generated plasmid pME4678 was digested with *Pme*I and the cassette (11374 bp) was used for transformation with AfS35, which resulted in AfGB143 after marker recycling.

**Plasmid and strain construction of *A. fumigatus* Δ*canA-N.*** The 3’ flanking region was amplified by oAMK286/283 (945 bp), which was ligated to *Pml*I linearized pCHS314. This plasmid was linearized with *Swa*I for ligation with the 5’ flanking region, which was amplified using oAMK290/291 (1492 bp) resulting in pME4681. AfS35 was transformed with the *Pme*I digested cassette (7692 bp) resulting in AfGB144 after marker recycling.

**Plasmid and strain construction of *A. fumigatus canA*^Δ838-4078^.** To delete only the *canA* sequence homolog to *A. nidulans candA-C* (base pairs 838-4078), the 5’ flanking region was amplified with oAMK295/296 (1203 bp) and ligated to the *Swa*I linearized plasmid containing the 3’ flanking region (oAMK267/268) resulting in pME4683. AfS35 and AfGB144 were transformed with *Pme*I digested cassette (7254 bp) resulting in AfGB145 and AfGB146, respectively after marker recycling.

**Plasmid and strain construction of *A. fumigatus mcherry:canA-N.*** For the construction a *canA-N* complementation plasmid with *mcherry:canA-N*, the 5’ flanking region of *canA-N* was amplified with oAMK290/310 (1492 bp), the *m-cherry* fragment was amplified with oAMK311/312 (753 bp) from gDNA of AGB1014 and *canA-N* with oAMK313/314 (1003 bp) from AfS35 gDNA. The fragments were ligated into the *Swa*I linearized vector containing the 3’ flanking region resulting in pME4801. AfGB144 was transformed with the *Pme*I digested cassette resulting in AfGB172 after marker recycling.

**Plasmid and strain construction of *A. nidulans* Δ*candA-N.*** For the *candA-N* deletion cassette, the 5‘ and 3’ flanking regions were amplified with oAMK61/111 (1772 bp) and oAMK112/79 (1528 bp), respectively. The fragments and the *nat*-RM were ligated with seamless cloning into linearized pUC19 provided by the kit. The resulting plasmid pME4650 was *Pme*I digested and the cassette (7905 bp) was transformed to AGB552. Marker recycling resulted in AGB1094.

**Plasmid and strain construction of *A. nidulans* Δ*candA-C.*** The *candA-C* deletion cassette was constructed by amplification of the 5‘ and 3’ flanking regions using oAMK87/109 (870 bp) and oAMK110/92 (552 bp), respectively. The fragments and the *nat-*RM were ligated with seamless cloning into linear pUC19 resulting in pME4651. The plasmid was *Pme*I digested and the cassette (6027 bp) was transformed to AGB552 and AGB1094. Marker recycling resulted in AGB1096 and AGB1098.

**Plasmid and strain construction of *A. nidulans candA-N* and *candA-C* complementation.** Complementation of *candA-N* was achieved by designing a flipper cassette containing the 5’ flanking region with *candA-N* amplified by oAMK61/137 (2852 bp) and oAMK112/79 (1528 bp) amplified 3’ flanking region. For *candA-C* complementation plasmid, 5’ flanking region and *candA-C* were amplified using oAMK87/138 (4124 bp) and the 3’ flanking region with oAMK110/92 (552 bp). The fragments for both complementation plasmids were ligated with the *phleo-RM* into pUC19 resulting in pME4655 and pME4656, respectively. Both were *Pme*I digested and the cassettes *candA-N* (9198 bp) and *candA-C* (9494 bp) were transformed to AGB1094 resulting in AGB1095 and to AGB1096 resulting in AGB1097 after marker recycling.

**Plasmid and strain construction of *A. nidulans* GFP-CandA-N.** For the construction of the N-terminal GFP fused CandA-N plasmid, the 5‘ flanking region of *candA-N* was amplified with oAMK61/94 (1772 bp). A fusion of *gfp* to a PreScission protease cleavage site (PP; sequence: LEVLFQGP) and to a linker (L; sequence: GGSGG) were amplified from pCM3 with EB10/oAMK95 (771 bp). *candA-N* was amplified with oAMK96/97 (1099 bp). The fragments were ligated by seamless cloning to linearized pUC19L. The resulting plasmid was linearized with *Swa*I, which restriction site was integrated by oAMK97. A non-recyclable marker cassette ^P^*gpdA-nat*^R^ was amplified from pME3929 using oAMK98/80 (1394 bp). 3‘ flanking *candA-N* was amplified with oAMK78/79 (1513 bp). The fragments were ligated with the linear vector from first cloning step resulting in pME4649. The plasmid was cut with *Pme*I and the cassette (6436 bp) was transformed with AGB989 resulting in AGB1103.

**Plasmid and strain construction of *A. nidulans* CandA-C-GFP.** C-terminal GFP tagged CandA-C was constructed by amplification of 5‘ flanking and *candA-C* with oAMK87/125 (4121 bp). A fusion of *PP:L:gfp:gpdA:nat* was amplified from pME4722 with oAMK82/80 (2127 bp). The 3‘ flanking *candA-C* was amplified with oAMK100/92 (552 bp). All fragments were ligated into linearized pUC19 with seamless cloning kit resulting in pME4652. The plasmid was *Pme*I digested and the cassette (6732 bp) was transformed to AGB989 resulting in AGB1104. pME4722 was constructed previously by amplification of a fragment (6081 bp) containing the 5‘ flanking *candA-N* with *candA-N:candA-C* fused genes (*candA-N* without stop codon, linked by *Spe*I site) from pME3310 using oAMK61/81. Prescission protease site (PP), linker (L) and *gfp* (777 bp)were amplified from pCM3 using oAMK82/85. Both fragments were cloned into pUC19 using the seamless cloning kit. The resulting plasmid was linearized with *Swa*I. A *gpdA:nat* fragment (1379 bp) was amplified from pME3929 with oAMK86/80. The 3‘ flanking *candA-N* (1513 bp) was amplified from wild type gDNA using oAMK78/79. Both fragments were cloned into the linearized plasmid using the seamless cloning kit resulting in pME4722.

**Plasmid and strain construction of *A. nidulans* Δ*candA-C1.*** Two different deletion cassettes for *candA-C1* were constructed, differing in the marker. For both the 5’ flanking region was amplified with oAMK142/127 (425 bp) and 3’ flanking regions with oAMK128/143 (307 bp). The fragments were ligated with either the *phleo-*RM or *ptrA-*RM into linearized pUC19 resulting in pME4653 and pME4657, respectively. *Pme*I restriction digest of pME4653 resulted in a 5553 bp deletion cassette, which was transformed to AGB552 leading to AGB1099 after marker recycling. *Pme*I digestion of pME4657 resulted in a deletion cassette of 5966 bp, which was transformed with AGB1103 and AGB1104 resulting in AGB1122 and AGB1123, respectively.

**Plasmid and strain construction of *A. nidulans candA-C1* complementation.** To complement *candA-C1*, a *gfp* fusion construct was designed. Therefore, *candA-C1* was amplified with its 5’ flanking region using oAMK126/131 (3400 bp), the *PP-L-gfp* fragment was amplified using oAMK82/ST06 (773 bp) and the 3’ flanking region with oAMK128/143 (307 bp). The fragments were ligated with *nat*-RM to linear pUC19 resulting in pME4658. The *Pme*I excised cassette with a size of 9070 bp was transformed to AGB1099 resulting in AGB1100 after marker recycling.

**Plasmid and strain construction of *A. nidulans* overexpression *candA-C-gfp.*** An overexpression *candA-C1* with C-terminal *gfp* fusion was achieved by amplification of *candA-C1* fused to *PP-L-gfp* and self-excisable *nat* flipper cassette and 3’ flanking region from pME4658 with Q5 polymerase (NEB) using oAMK224/143 (6280 bp). The fragment was ligated to pBluescript KS(+). The plasmid was linearized with *Swa*I, which restriction site was integrated within oAMK224. The 5’ flanking region of *candA-C1* was amplified by oAMK126/225 (2857 bp) and the nitrate promoter was amplified from pME4662 using oAMK226/227 (1281 bp). Both fragments were combined by fusion PCR using oAMK126/227 (4108 bp) and then ligated to the linearized plasmid resulting in pME4670. The cassette was excised with *Pme*I and AGB552, AGB1094 and AGB1096 were transformed with the fragment of 10340 bp resulting in AGB1101, AGB1129 and AGB1128 after marker recycling. To visualize nuclei in this strain, AGB1101 was transformed with pME3857 resulting in AGB1102. AGB1128 was transformed with the Δ*candA-N* flipper cassette obtained from pME4650 resulting in AGB1130 after marker recycling.

**Plasmid and strain construction of *A. nidulans* Δ*iORF.*** To delete the *intergenic region* (*iORF*) between *candA-C1* and *candA-C* open reading frames a deletion flipper cassette was designed. The 5’ flanking region was amplified with oAMK214/215 (879 bp) and the 3’ flanking region using oAMK216/217 (1005 bp). Both fragments were ligated together with a *nat-*RM into pUC19 producing the plasmid pME4668. AGB552 was transformed with the *Pme*I excised cassette of 6489 bp resulting in AGB1105 after marker recycling.

**Plasmid and strain construction of *A. nidulans* Δ*candA-C1/iORF.*** To generate a *candA-C1/iORF* double deletion cassette, the 5’ flanking was amplified with oAMK142/127 (428 bp) and the 3’ flanking region with oAMK216/217 (1005 bp). Both fragments were ligated together with a *nat*-RM into pUC19 giving the plasmid pME4669. AGB552 was transformed with the *Pme*I excised cassette of 6038 bp resulting in AGB1106 after marker recycling.

**Plasmid and strain construction of an *A. nidulans candA-C1:iORF:candA-C:gfp* fusion construct.** The *candA-C* 3’ flanking region was amplified using oAMK232/92 (537 bp). This fragment was ligated to *Swa*I linearized pME4696. oAMK243/228 were used for amplification of 5’ flanking region together with *candA-C1* (971 bp)*,* deleting its stop codon. The *iORF* was amplified using oAMK229/230 (299 bp). Both fragments were fused using oAMK243/230 (1240 bp). *candA-C* was amplified without its first ATG start codon together with *PP-L-gfp* from pME4652 oAMK231/EB2 were used (4022 bp). This fragment was fused to the PCR product of oAMK243/230 giving a product of 5232 bp. This fragment was then ligated to the *Pml*I digested plasmid containing the *candA-C* 3’ flanking region. The resulting plasmid was pME4671. AGB1096 was transformed with the *Pme*I digested fragment (10434 bp) resulting in AGB1110 after marker recycling.

**Plasmid and strain construction of an *A. nidulans candA-C1:candA-C-gfp* fusion construct.** The plasmid backbone and 5’ flanking region of *candA-C1* and *candA-C1* were amplified from pME4676 with oAMK298/299 generating a fragment of 7955 bp. *candA-C-PP-L-gfp* (cDNA) was amplified from pME4671 using oAMK03b/EB2 resulting in a fragment of 3882 bp. The 3’ flanking region of *candA-C* was amplified using oAMK300/92 giving a 537 bp fragment. The oAMK03/EB2 fragment was fused with the oAMK300/92 fragment by fusion PCR using oAMK03/92 resulting in a 4419 bp fusion product. This fragment was then ligated with the oAMK298/299 fragment resulting in pME4682. The plasmid was ectopically integrated into AGB1096 resulting in AGB1187.

**Plasmid and strain construction of *A. nidulans candA-C* with ΔATG.** To analyze if *candA-C1* translation is dependent on its predicted start codon, the predicted ATG start codon of *candA-C1* was deleted. The 5’ flanking region was amplified using oAMK142/255 (390 bp) and *candA-C1* was amplified without the first ATG using oAMK254/139 (543 bp). Both fragments were fused using oAMK142/139 (971 bp). The 3’ flanking region was amplified using oAMK128/143 (269 bp). All fragments were ligated with the *phleo-RM* into linear pUC19 resulting in the plasmid pME4674. AGB552 was transformed with *Pme*I excised cassette (6096 bp) resulting in AGB1108 after marker recycling.

**Plasmid and strain construction of *A. nidulans candA-C* NLS deletion strains.** The 3’ flanking region of *candA-C* was amplified with oAMK232/92 (537 bp) and ligated to *Pml*I linearized pCHS314. The 5’ flanking region was amplified together with one part of *candA-C* using oAMK87/276 (1461 bp) and the second half oAMK277/04b (2663 bp). Fusion of both fragments deleted the nuclear localization signal (NLS) sequence and the resulting fragment was ligated to *Swa*I linearized vector from the first cloning step resulting in pME4679. The *Pme*I excised cassette (9901 bp) was used for transformation with AGB1103 and AGB1102 and marker recycling resulted in AGB1125 and AGB1127, respectively. To study CandA-C localization without NLS the pME4652 plasmid was amplified with oAMK278/279 (9384 bp), deleting the NLS. Ligation resulted in pME4680. AGB1096 was transformed with *Pme*I cleaved cassette (6717 bp) and pME3173 for observing RFP fluorescence in the nuclei resulting in AGB1124.

**Plasmid and strain construction of *A. nidulans* Δ*candA-C1* complementation with *A. fumigatus canA exon 1.*** To analyze whether *A. fumigatus* *canA^exon1^* would complement *candA-C1* deletion we designed a complementation cassette. 5’ flanking *candA-C1* was amplified by oAMK142/256 (390 bp) and then fused to the PCR product of oAMK257/253, which is *Af_canA^exon1^* (596 bp)*.* The 3’ flanking region of *candA-C1* was amplified with oAMK128/143 (269 bp). Both fragments were ligated with the *phleo*-RM cassette to linear pUC19 resulting in pME4675. AGB552 was transformed with *Pme*I excised cassette (6149 bp) resulting in AGB1109 after marker recycling.

**Plasmid and strain construction of Δ*csnE.*** The 5’ flanking region was amplified using AL39/40 (903 bp) and the 3’ flanking region with AL47/48 (1295 bp). Ligation of flanking regions with the *phleo*-RM into pUC19 resulted in pME4654. The *Pme*I digested plasmid was a cassette of 7015 bp that was transformed to AGB552 creating AGB1111 after marker recycling. For double deletions with *candA-N* and *candA-C*, as well as a triple deletion with *candA-N/C*, AGB1094, AGB1096 and AGB1098 were transformed with the cassette resulting from pME4654 giving strains AGB1112, AGB1113 and AGB1114, respectively.

**Plasmid and strain construction for BiFC.** A BiFC cloning plasmid was used (pME4313) with bidirectional nitrate promoter. Either *Swa*I and *Pme*I restriction sites between each promoter and terminator allow insertion of two fragments fused to either *yfp^N^,* which expresses the N-terminal half of YFP or fused to *yfp^C^* expressing the C-terminal half of YFP*.* To generate the preBiFCII plasmid (pME4684) *yfp^N^* was amplified from pME3741 with oAMK173/163 (465 bp) and ligated to *Swa*I linearized pME4313 resulting in pME4684. To generate preBiFCIII cloning plasmid containing *candA-C1-L-yfp^N^,* *yfp^N^-L* (linker: RSIAT) was amplified using oAMK198/CM108 and *candA-C1* using oAMK197/120. Both fragments were fused using oAMK199/200 for ligation to *Swa*I linearized pME4313 resulting in pME4685.

**Plasmid and strain construction of *candA-N, candA-C* and *candA-C1* BiFC control plasmids.** oAMK168b/169b were used to amplify *yfp^C^-L* (linker: RSIATGAP) from pME3741 and oAMK170/02 for *candA-N*. Both fragments were fused (1362 bp). Primer oAMK03b/167 were used to amplify *candA-C-L-yfp^C^* (3441 bp) from pME3741. pME4684 was linearized with *Pme*I and used for ligation with *yfp^C^-L-candA-N* or *candA-C-L-yfp^C^* resulting in pME4662 and pME4663, respectively. *yfp^C^* was amplified from pME3741 with oAMK168b/167 and ligated to *Pme*I linearized pME4685 resulting in pME4666. Transformation of AGB1014 with pME4662 resulted in AGB1115, with pME4663 in AGB1116 and with pME4666 resulting in AGB1119. *candA-C1* was amplified with oAMK120/204 producing a fragment of 558 bp. *yfp^C^* was amplified with oAMK205/167 from pME3741, which resulted in a fragment of 333 bp. Both fragments were fused by fusion PCR using oAMK120/167 giving a fragment of 861 bp. This fragment was ligated into *Pml*I linearized pME4684 resulting in pME4719. AGB1014 was transformed with pME4719 resulting in AGB1119.

**Plasmid and strain construction of CandA-C1 BiFC with CandA-N or CandA-C.** For interaction studies of CandA-C1 with CandA-N and CandA-C, PCR fragments of oAMK03b/167 (*candA-C-L-yfp^C^*), oAMK168b/02 (*yfp^C^-L-candA-N*) and were ligated in *Pme*I linearized pME4685 resulting in pME4664 and pME4665, respectively. AGB1014 was transformed with pME4664 resulting in AGB1117 and with pME4665 in resulting in AGB1118. For BiFC experiments in a *csnE* deletion strain AGB1111 was transformed with pME4664 and pME4665 resulting in AGB1120 and AGB1121, respectively.

**Plasmid and strain construction of CandA-N~A-C1~A-C~HA fusion.** A fusion of all three CandA subunits was constructed. The fragment containing the 5’ flanking region of *candA-C1*, 3’*candA-C* and the *nat* flipper cassette were amplified from pME4671 with oAMK315/316 (8627 bp). This fragment was used as a backbone. The *candA-N* fragment was amplified with oAMK317/318 (969 bp) from AGB552 cDNA. *candA-C1* was amplified together with *candA-C* from pME4682 using oAMK120/319 (3696 bp), integrating a HA-tag sequence and the fragment was again amplified with oAMK120/320 for the seamless cloning reaction of both fragments into the backbone resulting in pME4802. The *Pme*I digested cassette was transformed into AGB1094 resulting in AGB1220 after marker recycling.

**Plasmid construction of pME4696.** The plasmid pBluescript KS was amplified with primers flip-1 and flip-2, which contained a *Pml*I or *Swa*I restriction site in their overhang, respectively. The linear PCR fragment and the *Sfi*I digested *nat*RM from plasmid pME4304 were ligated in a Seamless cloning reaction. The resulting plasmid served as cloning vector for further constructs.

***In vitro* protein pull-down.** Mycelia for pull-down experiments was obtained from 1 l submerged cultures inoculated with 2x10^6^ spores for 20 hours at 37°C on a rotary shaker. The crude extracts were prepared as described in materials and methods. The B* buffer mixed mycelium was centrifuged for 1 hour at 4°C and 15 000 rpm in a Sorvall RC-5B Plus superspeed centrifuge (GMI). The supernatant was filtered through miracloth (MILLIPORE) into a polyprep column (BIORAD) containing 80 µL GFP-Trap® beads (CHROMOTEK) pre-equilibrated with B* buffer. After incubation of the supernatant with GFP beads on a rotary shaker for 2 hours at 4°C the beads were washed with 6 ml washing buffer W300 (300 mM NaCl, 10 mM Tris pH 7.5, 0.5 mM EDTA) and W500 (500 mM NaCl, 10 mM Tris pH 7.5, 0.5 mM EDTA). Elution of GFP-trap beads bound proteins was performed three times by adding 0.2 M glycine pH 2.5 for 25 seconds and then neutralization with 1 M Tris pH 10.4. Elution samples were mixed with protein sample buffer (250 mM Tris-HCl pH 6.8, 15 % (v/v) 2-Mercaptoethanol, 30 % (v/v) Glycerol, 7 % (w/v) SDS, 0.3 % (w/v) Bromphenolblue) and then boiled at 95°C for 5 min for SDS page analysis. This was followed by bottom-up protein analysis with mass spectrometry after pull-down experiments.

***In-gel* digest of proteins with trypsin.** For the analysis of proteins with LC-MS, samples were subjected to SDS-PAGE and shortly run into the resolving gel. Proteins were stained with Colloidal blue Coomassie solution overnight. Gel-lanes were cut into small pieces of approximately 2 mm^2^ in size for *in-gel* digestion with trypsin according to the protocol of (14) followed by stage tip purification as described previously (10, 15–17).

**Peptide analysis with LC-MS**. Peptide samples were analyzed with mass spectrometry coupled to liquid chromatography (LC-MS). Therefore, an Orbitrap Velos Pro^TM^ mass spectrometer and an Ultimate 3000^TM^ liquid chromatography system (both THERMO FISHER SCIENTIFIC) were used. Peptides were separated at nano-flow rates (300 nl/min) using Acclaim PepMap RSLC^TM^ columns (THERMO FISHER SCIENTIFIC) through the application of a water-acetonitrile gradient. Chromatographically separated peptides were on-line ionized by electrospray (nESI) using the Nanospray Flex Ion Source^TM^ (THERMO FISHER SCIENTIFIC) at 2.4 kV, and continuously transferred into the mass spectrometer. Full scans within the mass range of 300-1850 were recorded with the Orbitrap-FT analyzer at a resolution of 30.000. In parallel data-dependent top-ten fragmentation spectra (MS2) were acquired by collision-induced dissociation (CID) in the LTQ Velos Pro^TM^ linear ion trap. The *XCalibur 2.2^TM^* software (THERMO FISHER SCIENTIFIC) was applied for LC-MS method programming and data acquisition. MS2 data processing for protein analysis and identification was carried out with the *MaxQuant* software and *Perseus 1.6.0.7* software (18, 19) or the *Proteome Discoverer 1.4^TM^* software (THERMO FISHER SCIENTIFIC) employing the *SequestHT^TM^* and *Mascot^TM^* search engines. As protein database an *A. nidulans-* and an *A. fumigatus-*specific database with common contaminants were used. Peptides of 1 µl sample solution were loaded with 0.07% TFA on an Acclaim® PepMap 100 pre-column (100 µm x 2 cm, C18, 3 µm, 100 Å, Thermo Scientific) at a flow rate of 20 µl/min for 3 min. Analytical peptide separation by reverse phase chromatography was performed at a flow rate of 300 nl/min on an Acclaim® PepMap RSLC column (75 µm x 50 cm, C18, 3 µm, 100 Å, THERMO FISHER SCIENTIFIC). A gradient from 98% solvent A (0.1% formic acid) and 2% solvent B (80% acetonitrile, 0.1% formic acid) to 55% B was applied within 30 min and was followed by 90% B for 4 min (Optima® LC-MS solvents and acids were purchased from Fisher Chemical). Nano ESI mass spectrometry – Q Exactive HF (THERMO FISHER SCIENTIFIC): Chromatographically eluting peptides were on-line ionized by nano-electrospray (nESI) using the Nanospray Flex Ion Source (THERMO FISHER SCIENTIFIC) at 1.5 kV (liquid junction) and continuously transferred into the mass spectrometer. Full scans within the mass range of 300-1,800 m/z were taken from the Orbitrap-FT analyzer at a resolution of 30,000 with parallel data-dependent top 10 MS2-fragmentation (HCD). The resolution was set to 60,000 for tSIM scans and to 15,000 for dd-MS2 scans. The maximum ion time was 100 ms for tSIM scans (AGC target 1e6) and 2,000 ms for dd-MS2 (AGC target 1e5). The loop count equaled the number of m/z values on the inclusion list. LC-MS method programming and data acquisition was performed with the software *XCalibur 4.0* (THERMO FISHER SCIENTIFIC).

**Perseus workflow for protein analysis and identification.** Raw data were analyzed with MaxQuant. Subsequently, the protein groups text file was uploaded to *Perseus 1.6.0.7* software for step-by-step processing of the MS2 data. Therefore, a *generic matrix upload* was performed and the following criteria were selected: Main: LFQ intensity; Numerical: unique peptides, MS/MS count; Categorical: only identified by site, reverse, potential contaminant; Text: protein IDs. Originally 1980 proteins were identified. The list was reduced to 1876 candidates by *filter rows based on categorical column* applied for only identified by site; reverse and potential contaminant. The *processing* command was used to remove empty columns. The LFQ intensities were logarithmized (log2(x)) using the *transformation* command. *Add annotation* was applied to annotate the proteins according to UniProt data base of *Aspergillus nidulans* FGSC A3_ATCC38163. Then, *categorical annotation rows* were introduced by creating Group 1 containing the log2(x) LFQ intensities of each pull-down replicate with the identical name (LFQ intensity AC, LFQ intensity AN, etc.). Missing values were preplaced by NaN using the *processing* command. The next filtering step included *filter rows based on valid values* with the criteria of min. three values in at least one group (Group 1), and the values should be valid. The matrix was reduced to 645 proteins. The missing values were replaced from normal distribution according to program settings (width = 0.3, down shift = 1.8, mode = separately for each column) using again the *processing* function. *Filter rows based on valid values* applying a min. number of three values in at least one group (Group 1), and the log2(x) LFQ values should be greater or equal 18 reduced the matrix to 198 proteins. *Categorical annotation rows* was performed to create group LFQ C, containing only the pull-down control columns. The matrix was reduced to 65 proteins by f*ilter rows based on valid values* applying a min. number of three values in at least one group (LFQ C), and log2(x) LFQ values should be less or equal 18. The column type of unique peptides was changed from numerical to main using the *processing* function. *Categorical annotation rows* created the group UP, which contains all unique peptide columns. *Filter rows based on valid values* applying a min. number of three values in at least one group (UP), and values should be greater or equal three reduced the matrix to 63 proteins. The column type of MS/MS count was changed from numerical to main using the *processing* function. The group MS/MS, containing all MS/MS count columns was created by the command c*ategorical annotation rows. Filter rows based on valid values* was performed with a min. number of three values in at least one group (MS/MS), and values should be less or equal three. The number of proteins did not change. Then the matrix was exported to excel applying the *generic matrix export* function. In Excel the proteins were sorted according to their cellular localization and function (using AspGD, UniProt and KEGG databases), and proteins which have more than one log2(x) LFQ value above 18 in the control were manually deleted. The excel file was saved as .txt file, which allowed a *generic matrix upload* to *Perseus,* with the following criteria: Main: LFQ intensity; Text: Gene names, Protein, Localization, Pathway. Applying the *hierarchical clustering* command allows the generation of a heat map showing the log2(x) LFQ intensity AN, log2(x) LFQ intensity AC, log2(x) LFQ intensity AC1, log2(x) LFQ intensity control. The log2(x) LFQ intensity color code was set to 12-15: black, 15-18: blue, 18-26: gradient from red to orange to yellow. After all these processing steps 51 valid enrichment candidates were left, which are listed in Tab S1. Top hits are depicted in the heat map of Fig 4.

**Thin layer chromatography.** Secondary metabolite extracts from seven days asexually and sexually developed *A. nidulans* strains (for extraction see Secondary metabolite extraction) were resuspended in 50 µl ethyl acetate. The experiment was conducted as described in (2). Twelve microliters were spotted on the TLC silica gel plates. The TLC was photographed at 366 nm with a Camag TLC Visualizer 2 system (Camag).

**Supplementary References**

1. Stajich JE, Harris T, Brunk BP, Brestelli J, Fischer S, Harb OS, Kissinger JC, Li W, Nayak V, Pinney DF, Stoeckert CJ, Roos DS, Roos DS. 2012. FungiDB: an integrated functional genomics database for fungi. Nucleic Acids Res 40:D675-81.

2. Yashavantha Rao HC, Rakshith D, Harini BP, Gurudatt DM, Satish S. 2017. Chemogenomics driven discovery of endogenous polyketide anti-infective compounds from endosymbiotic *Emericella variecolor CLB38* and their RNA secondary structure analysis. PLoS One 12:e0172848.

3. Jun Han S, Jae Lee B, Sam Kang H. 1998. Purification and characterization of the nuclear ribonuclease P of *Aspergillus nidulans*. Eur J Biochem 251:244–251.

4. Walker SC, Engelke DR. 2006. Ribonuclease P: the evolution of an ancient RNA enzyme. Crit Rev Biochem Mol Biol 41:77–102.

5. Hartmann T, Dümig M, Jaber BM, Szewczyk E, Olbermann P, Morschhäuser J, Krappmann S. 2010. Validation of a self-excising marker in the human pathogen *Aspergillus fumigatus* by employing the beta-rec/six site-specific recombination system. Appl Environ Microbiol 76:6313–7.

6. Bayram Ö, Krappmann S, Ni M, Bok JW, Helmstaedt K, Valerius O, Braus-Stromeyer S, Kwon N-J, Keller NP, Yu J-H, Braus GH. 2008. VelB/VeA/LaeA complex coordinates light signal with fungal development and secondary metabolism. Science 320:1504–6.

7. Helmstaedt K, Schwier EU, Christmann M, Nahlik K, Westermann M, Harting R, Grond S, Busch S, Braus GH. 2011. Recruitment of the inhibitor Cand1 to the cullin substrate adaptor site mediates interaction to the neddylation site. Mol Biol Cell 22:153–164.

8. Bayram Ö, Bayram ÖS, Ahmed YL, Maruyama J, Valerius O, Rizzoli SO, Ficner R, Irniger S, Braus GH. 2012. The *Aspergillus nidulans* MAPK Module AnSte11-Ste50-Ste7-Fus3 Controls Development and Secondary Metabolism. PLoS Genet 8:e1002816.

9. Christmann M, Schmaler T, Gordon C, Huang X, Bayram O, Schinke J, Stumpf S, Dubiel W, Braus GH. 2013. Control of multicellular development by the physically interacting deneddylases DEN1/DenA and COP9 signalosome. PLoS Genet 9:e1003275.

10. Thieme KG, Gerke J, Sasse C, Valerius O, Thieme S, Karimi R, Heinrich AK, Finkernagel F, Smith K, Bode HB, Freitag M, Ram AFJ, Braus GH. 2018. Velvet domain protein VosA represses the zinc cluster transcription factor SclB regulatory network for *Aspergillus nidulans* asexual development, oxidative stress response and secondary metabolism. PLOS Genet 14:e1007511.

11. Krappmann S, Sasse C, Braus GH. 2006. Gene targeting in *Aspergillus fumigatus* by homologous recombination is facilitated in a nonhomologous end- joining-deficient genetic background. Eukaryot Cell 5:212–5.

12. Lin C-J, Sasse C, Gerke J, Valerius O, Irmer H, Frauendorf H, Heinekamp T, Straßburger M, Tran VT, Herzog B, Braus-Stromeyer SA, Braus GH. 2015. Transcription factor SomA is required for adhesion, development and virulence of the human pathogen *Aspergillus fumigatus*. PLoS Pathog 11:e1005205.

13. Kolog Gulko M, Heinrich G, Gross C, Popova B, Valerius O, Neumann P, Ficner R, Braus GH. 2018. Sem1 links proteasome stability and specificity to multicellular development. PLOS Genet 14:e1007141.

14. Shevchenko A, Wilm M, Vorm O, Mann M. 1996. Mass spectrometric sequencing of proteins from silver-stained polyacrylamide gels. Anal Chem 68:850–858.

15. Opitz N, Schmitt K, Hofer-Pretz V, Neumann B, Krebber H, Braus GH, Valerius O. 2017. Capturing the Asc1p/receptor for activated C kinase 1 (RACK1) microenvironment at the head region of the 40S ribosome with quantitative BioID in yeast. Mol Cell Proteomics 16:2199–2218.

16. Schmitt K, Smolinski N, Neumann P, Schmaul S, Hofer-Pretz V, Braus GH, Valerius O. 2017. Asc1p/RACK1 connects ribosomes to eukaryotic phosphosignaling. Mol Cell Biol 37:e00279-16.

17. Rappsilber J, Mann M, Ishihama Y. 2007. Protocol for micro-purification, enrichment, pre-fractionation and storage of peptides for proteomics using StageTips. Nat Protoc 2:1896–1906.

18. Cox J, Mann M. 2008. MaxQuant enables high peptide identification rates, individualized p.p.b.-range mass accuracies and proteome-wide protein quantification. Nat Biotechnol 26:1367–1372.

19. Tyanova S, Temu T, Sinitcyn P, Carlson A, Hein MY, Geiger T, Mann M, Cox J. 2016. The Perseus computational platform for comprehensive analysis of (prote)omics data. Nat Methods 13:731–740.
