# Supplementary material for: Integration of Fungus-Specific CandA-C1 into a Trimeric CandA Complex Allowed Splitting of the Gene for the Conserved Receptor Exchange Factor of CullinA E3 Ubiquitin Ligases in Aspergilli
Source: mBio. 2019 Jun 18;10(3):e01094-19. doi: 10.1128/mBio.01094-19 (PMC6581859; doi:10.1128/mBio.01094-19)
Supplement: TABLE S3 [file mBio.01094-19-st003.docx]

**TABLE S3** Oligonucleotides designed and used in this study, p.c. = personal communication.

| Primer | Sequence (5’ to 3’) | Reference |
| --- | --- | --- |
| AL39 | ATTCGAGCTCGGTACGTTTAAACACTTACTCGTCCACAAGCTT | This study |
| AL40 | ACCTATAGGCCTGAGTGATGATTGTCAGGTGGGGATAT | This study |
| AL47 | AAGTTGAGCATAATATCAGATGATGAGACGATCTATG | This study |
| AL48 | CCAAGCTTGCATGCCGTTTAAACTGGGGACGATATGATCAG | This study |
| CM108 | CGCTCCATCGCCACGGTGAGCAAGGGCGAGGA | C. Meister |
| EB2 | CTACTTGTACAGTTCGTCCATG | E. Beckmann, p.c. |
| EB10 | ATGGTGAGCAAGGGCGAGG | E. Beckmann, p.c. |
| flip-1 | ACCTATAGGCCTGAGATTTAAATATCGAATTCCTGCAGCCCGG | C. Sasse p.c. |
| flip-2 | ATAATATGGCCATCTCACGTGATCAAGCTTATCGATACCGTCG | C. Sasse p.c. |
| MG277-RT *h2A-f* | CGTCTTCTTCGCAAGGGAAACT | (13) |
| MG278-RT *h2A-r* | CGGGTTTTCTTGTTGTCACGAG | (13) |
| oAMK01 | ATGGGAGATCGACACAC | This study |
| oAMK02 | CTACATACCAGTAATTAGCTTTTCTTTAGGC | This study |
| oAMK03b | ATGTCTTCCGACGCAATGTCG | This study |
| oAMK04b | TTAGAACTCCGACTCGAGGTTA | This study |
| oAMK85 | CCAAGCTTGCATGCCATTTAAATCTACTTGTACAGTTCGTCCATG | This study |
| oAMK86 | GAACTGTACAAGTAGATTTGGCGGCTCTGAGGTGCAG | This study |
| oAMK87 | ATTCGAGCTCGGTACGTTTAAACTAATATCTCAGTCAAAGATGCATTTC | This study |
| oAMK92 | CCAAGCTTGCATGCCGTTTAAACTCATTTGATTCTTTTGATTACCC | This study |
| oAMK94 | GCCCTTGCTCACCATCGTGAACAGAACCCCGCG | This study |
| oAMK95 | ACCACCGCTACCACCGGG | This study |
| oAMK96 | GGTGGTAGCGGTGGTATGGGAGATCGACACACGATC | This study |
| oAMK97 | CCAAGCTTGCATGCCATTTAAATCTACATACCAGTAATTAGCTTTTC | This study |
| oAMK98 | CTGGTATGTAGATTTGGCGGCTCTGAGGTGCAG | This study |
| oAMK100 | ATGCCCTGCCCCTGAGACAAGACTTTGCCATTGGATTT | This study |
| oAMK109 | CCTATAGGCCTGAGTTGTGGCGGGTGAGTGAGTT | This study |
| oAMK111 | CCTATAGGCCTGAGTCGTGAACAGAACCCCGCG | This study |
| oAMK110 | ATAATATGGCCATCTGACAAGACTTTGCCATTGG | This study |
| oAMK112 | ATAATATGGCCATCTTCTAGCATTTATTTATGGGCTGG | This study |
| oAMK120 | ATGCATTTCAAAGAACACACCCC | This study |
| oAMK121 | TCACTGGAGAAAGTCGAAC | This study |
| oAMK125 | GAACAGAACTTCCAGGAACTCCGACTCGAGGTTAC | This study |
| oAMK126 | ATTCGAGCTCGGTACGTTTAAACACACCTGCTATAGCAAGC | This study |
| oAMK127 | CCTATAGGCCTGAGTCTTTGACTGAGATATTAAGACGAG | This study |
| oAMK128 | ATAATATGGCCATCTTTTTACCATTACTGCATTATCTTCG | This study |
| oAMK131 | GAACAGAACTTCCAGCTGGAGAAAGTCGAACAGATCA | This study |
| oAMK137 | CCTATAGGCCTGAGTCTACATACCAGTAATTAGCTTTTC | This study |
| oAMK138 | CCTATAGGCCTGAGTTTAGAACTCCGACTCGAGGTTA | This study |
| oAMK139 | CCTATAGGCCTGAGTTCACTGGAGAAAGTCGAAC | This study |
| oAMK142 | ATTCGAGCTCGGTACGTTTAAACAATGACTTATCAAAGCCAGAGGC | This study |
| oAMk143 | CCAAGCTTGCATGCCGTTTAAACTGTGGCGGGTGAGTGAGT | This study |
| oAMK163 | ATGGTGAGCAAGGGCGAG | This study |
| oAMK167 | TTACTTGTACAGCTCGTCCATG | This study |
| oAMK168b | ATGGCCGACAAGCAGAAGAACG | This study |
| oAMK169b | CGGCGCGCCCGTGGCGATGGAGCGCTTGTACAGCTCGTCCATGC | This study |
| oAMK170 | GCCACGGGCGCGCCGATGGGAGATCGACACACGATC | This study |
| oAMK173 | CAGCCACAACGTCTATATCATGTAG | This study |
| oAMK197 | CGTGGCGATGGAGCGCTGGAGAAAGTCGAACAGATC | This study |
| oAMK198 | CTACATGATATAGACGTTGTGGC | This study |
| oAMK199 | TGCGAACCCGTATTTCTACATGATATAGACGTTGTGGC | This study |
| oAMK200 | CATACTCTCACATTTATGCATTTCAAAGAACACACCCC | This study |
| oAMK204 | CTTGCAGGCCGGGCGCTGGAGAAAGTCGAACAGATC | This study |
| oAMK205 | TTCGACTTTCTCCAGCGCCCGGCCTGCAAGATC | This study |
| oAMK214 | ATTCGAGCTCGGTACGTTTAAACCTTCTCAGAACAACATGAC | This study |
| oAMK215 | CCTATAGGCCTGAGTTCACTGGAGAAAGTCGAACAGAT | This study |
| oAMK216 | ATAATATGGCCATCTATGTCTTCCGACGCAATGTCG | This study |
| oAMK217 | CCAAGCTTGCATGCCGTTTAAACTCAGGGTTTCAATCTGCAG | This study |
| oAMK224 | CGGTACATTTAAATATGCATTTCAAAGAACACACCCC | This study |
| oAMK225 | ATCCCATACTCTCACCTTTGACTGAGATATTAAGACGAG | This study |
| oAMK226 | ATATCTCAGTCAAAGGTGAGAGTATGGGATAGGAAAAT | This study |
| oAMK227 | GATGGCGGGCGCGGTGAT | This study |
| oAMK228 | ATAATATGGCCATCTTTTTACCATTACTGCATTATCTTCG | This study |
| oAMK229 | TTCGACTTTCTCCAGTTTTACCATTACTGCATTATCTTCG | This study |
| oAMK230 | CATTGCGTCGGAAGATGTGGCGGGTGAGTGAGTT | This study |
| oAMK231 | CACTCACCCGCCACATCTTCCGACGCAATGTCGG | This study |
| oAMK232 | GACAAGACTTTGCCATTGGATTT | This study |
| oAMK243 | ATTCGAGCTCGGTACGTTTAAAC | This study |
| oAMK247 | ATTCGAGCTCGGTACGTTTAAACTTTCGGGTTGAGAGAAGC | This study |
| oAMK248 | CCTATAGGCCTGAGTATCGTCAAAAGTAAAAGGGCTGC | This study |
| oAMK249 | ATAATATGGCCATCTGTGAGCTACCTCGGGGGC | This study |
| oAMK250 | CCAAGCTTGCATGCCGTTTAAACCATCCAAGATAATGGGCATGTA | This study |
| oAMK252 | TTTACTTTTGACGATATGCATTTCAAAGAACACACC | This study |
| oAMK253 | CCTATAGGCCTGAGTTGCTGTAGAAAATCGAATAAATCAAG | This study |
| oAMK254 | ATATCTCAGTCAAAGCATTTCAAAGAACACACCCCTC | This study |
| oAMK255 | GTGTTCTTTGAAATGCTTTGACTGAGATATTAAGACGAG | This study |
| oAMK256 | ATCACTGATAGGCATCTTTGACTGAGATATTAAGACGAG | This study |
| oAMK257 | ATATCTCAGTCAAAGATGCCTATCAGTGATCATACTCC | This study |
| oAMK262 | TTCTTTGAAATGCATATCGTCAAAAGTAAAAGGGCTGC | This study |
| oAMK267 | ATATGGCCATCTCACGCGTGCCTTTTAGACGAAAGAA | This study |
| oAMK268 | GATAAGCTTGATCACGTTTAAACTGGAGCAACTCGGCGTGC | This study |
| oAMK269 | AGGAATTCGATATTTGTTTAAACTTACGCAACCCTCAAAAGC | This study |
| oAMK270 | GAACAGAACTTCCAGCTAGAATTCCGTCTCGAGGC | This study |
| oAMK271 | ATAGGCCTGAGATTTTACTTGTACAGTTCGTCCATGC | This study |
| oAMK273 | AGGAATTCGATATTTGTTTAAACTTTCGGGTTGAGAGAAGC | This study |
| oAMK274 | ATAGGCCTGAGATTTATCGTCAAAAGTAAAAGGGCTGC | This study |
| oAMK276 | GGCATCGCTATCTTGCGAGATCTTGGAACCACTAC | This study |
| oAMK277 | GGTTCCAAGATCTCGCAAGATAGCGATGCCAGTATG | This study |
| oAMK278 | CGAGATCTTGGAACCACTAC | This study |
| oAMK279 | CAAGATAGCGATGCCAGTATG | This study |
| oAMK283 | CCAAGCTTGCATGCCGTTTAAACAACGGTTAGACTGAGCACA | This study |
| oAMK286 | GTATACCCATTGAATGACGTGTC | This study |
| oAMK290 | AGGAATTCGATATTTGTTTAAACTATTGTGCTATGTAATATATTAC | This study |
| oAMK291 | ATAGGCCTGAGATTTGAGGACGTTGAAGTTCGAAAA | This study |
| oAMK295 | GTTTAAACACGTGCAAAGATGGATGTG | This study |
| oAMK296 | CTAGCTGATTGTGTTTAGACACT | This study |
| oAMK298 | CTGGAGAAAGTCGAACAGATC | This study |
| oAMK299 | ATCAAGCTTATCGATACCGTC | This study |
| oAMK300 | GAACTGTACAAGTAGGACAAGACTTTGCCATTGGATTT | This study |
| oAMK310 | GCCCTTGCTGACCATGAGGACGTTGAAGTTCGAA | This study |
| oAMK311 | ATGGTCAGCAAGGGCGAAG | This study |
| oAMK312 | ACCACCGCTACCACCCTTGTACAGCTCGTCCATGC | This study |
| oAMK313 | GGTGGTAGCGGTGGTATGGCGGACAGACAGGCC | This study |
| oAMK314 | ATAGGCCTGAGATTTTCAAGCTACCCGACCAAGAC | This study |
| oAMK315 | CTTTGACTGAGATATTAAGACGAG | This study |
| oAMK316 | GTGAGATGGCCATATTATAG | This study |
| oAMK317 | ATATCTCAGTCAAAGATGGGAGATCGACACAC | This study |
| oAMK318 | TTCTTTGAAATGCATGGCAACTGACGGTATTGG | This study |
| oAMK319 | CTAGGCGTAGTCGGGGACGTCGTAGGGGTAGAACTCCGACTCGAGGTTA | This study |
| oAMK320 | ATATGGCCATCTCACCTAGGCGTAGTCGGGGAC | This study |
| rtAMK01_*candA-C1-f* | AGACGGGGCTACTGTCTACAAG | This study |
| rtAMK02_*candA-C1-r* | GACTTGAGCAGGCTGAGTTTTT | This study |
| rtAMK03_*candA-C-f* | CTCCATTCCGTCAAGGAGTTAC | This study |
| rtAMK04_*candA-C-r* | CTCCCATAGCACGGTTATCTTC | This study |
| rtAMK05_*candA-N-f* | GTATTCTCAGCAATCCCTGCTC | This study |
| rtAMK06_*candA-N-r* | AGACATTTCAGTGCCTGGTTTT | This study |
| ST06 | cctataggcctgagtCTACTTGTACAGTTCGTCCATG | C. Meister |
