# Supplementary material for: Integration of Fungus-Specific CandA-C1 into a Trimeric CandA Complex Allowed Splitting of the Gene for the Conserved Receptor Exchange Factor of CullinA E3 Ubiquitin Ligases in Aspergilli
Source: mBio. 2019 Jun 18;10(3):e01094-19. doi: 10.1128/mBio.01094-19 (PMC6581859; doi:10.1128/mBio.01094-19)
Supplement: TABLE S4 [file mBio.01094-19-st004.docx]

**TABLE S4 Plasmids designed and used in this study.** *A. nidulans* genes are labeled with An_geneX and *A. fumigatus* genes with Af_geneX. ^P^: promoter, ^T^: terminator, ^R^: resistance, PP: PreScission cleavage site; L: linker, *bla:*ampicillin resistance gene, *nat*-RM: recyclable *nat* resistance cassette from pME4304 or pME4696, *phleo-*RM: recyclable phleo resistance cassette from pME4305, *ptrA-*RM: recyclable *ptrA* resistance cassette from pSK485 or pCHS314, p.c. = personal communication.

| Plasmid | Genotype | Reference |
| --- | --- | --- |
| pBluescript  KSII-/+ | cloning vector | FERMENTAS |
| pCHS314 | Cloning plasmid with self-excising *β-rec/six* *ptrA-*RM | C. Sasse, p.c. |
| pCM3 | Template for PCRs; contains *gfp:L:PP:csnF* | C. Meister |
| pME3173 | Plasmid for ectopic integration of *mrfp:h2A:nat*^R^ | (6) |
| pME3310 | *candA-N:candA-C* fusion, used as template for PCR amplifications | (7) |
| pME3741 | BiFC vector, used as template for PCR amplifications | (7) |
| pME3857 | Plasmid for ectopic integration of *mrfp:h2A:phleo*^R^ | (8) |
| pME3929 | *gfp:natR* cassette, used as template for PCR | (9) |
| pME4304 | self-excising *β-rec/six* *nat*-RM containing vector | (10) |
| pME4305 | self-excising *β-rec/six* *phleo-*RM containing vector | (10) |
| pME4313 | pME3741 BiFC cloning plasmid | (10) |
| pME4649 | *^P^candA-N:gfp:L:PP:An_candA-N:^P^gpdA:nat^R^:candA-N^T^* in pUC19, *bla* | This study |
| pME4650 | An_Δ*candA-N:nat*-RM: in pUC19, *bla* | This study |
| pME4651 | An_Δ*candA-C:nat*-RM in pUC19, *bla* | This study |
| pME4652 | *^P^candA-C:An_candA-C:PP:L:gfp:gpdA:nat^R^:candA-C^T^* in pUC19, *bla* | This study |
| pME4653 | An_Δ*candA-C1:phelo-*RM in pUC19 | This study |
| pME4654 | An_Δ*csnE:phleo-RM* in pUC19, *bla* | This study |
| pME4655 | Complementation An_*candA-N:phelo-*RM in pUC19, *bla* | This study |
| pME4656 | Complementation An_*candA-C:phelo-*RM in pUC19, *bla* | This study |
| pME4657 | An_Δ*candA-C1:ptrA-RM* in pUC19, *bla* | This study |
| pME4658 | Complementation An_*candA-C1:nat-*RM in pUC19, *bla* | This study |
| pME4662 | BiFC vector *^T^NiiA:yfp^N^:^P^niiA/niaD:yfp^C^:*An_*candA-N:NiiD^T^:phleo^R^* in pME3741, *bla* | This study |
| pME4663 | BiFC vector *^T^NiiA:yfp^N^:^P^niiA/niaD:*An_*candA-C:yfp^C^:NiiD^T^:phleo^R^* in pME3741, *bla* | This study |
| pME4664 | BiFC vector *^T^NiiA:yfp^N^:*An_*candA-C1:^P^niiA/niaD:*An_*candA-C:yfp^C^:NiiD^T^:phleo^R^* in pME3741, *bla* | This study |
| pME4665 | BiFC vector *^T^NiiA:yfp^N^:*An_*candA-C1:^P^niiA/niaD:yfp^C^:*An_*candA-N:NiiD^T^:phleo^R^* in pME3741, *bla* | This study |
| pME4666 | BiFC vector *^T^NiiA:yfp^N^:*An_*candA-C1:^P^niiA/niaD:yfp^C^:NiiD^T^:phleo^R^* in pME3741, *bla* | This study |
| pME4668 | An_Δ*iORF:phleo-*RM in pUC19, *bla* | This study |
| pME4669 | An_Δ*candA-C1*/*iORF:phleo-*RM in pUC19, *bla* | This study |
| pME4670 | Overexpression An_*candA-C1-gfp:nat-*RM in pBluescriptKS(‑), *bla* | This study |
| pME4671 | Fusion of An_*candA-C1:candA-C:nat*-RM in pME4696, *bla* | This study |
| pME4672 | Af_*canA*^Δ^*^exon1^:ptrA-*RM in pUC19, *bla* | This study |
| pME4673 | Complementation of Af_*canA*^Δ^*^exon1^* with  An_*candA-C1:ptrA-*RM in pUC19, *bla* | This study |
| pME4674 | An_*candA-C1* start codon deletion:*phleo-*RM in pUC19, *bla* | This study |
| pME4675 | Complementation An_ Δ*candA-C1* with  Af_*canA*^Δ^*^exon1^*:*phleo-*RM in pUC19, *bla* | This study |
| pME4676 | Plasmid for ectopic integration of *candA-C1* | This study |
| pME4677 | Af_*ΔcanA:ptrA-*RM in pUC19, *bla* | This study |
| pME4678 | Complementation Af_*canA:ptrA-*RM in pUC19, *bla* | This study |
| pME4679 | An_*candA-C*^Δ^*^NLS^:ptrA-*RM in pCHS314, *bla* | This study |
| pME4680 | *^P^candA-C:*An_*candA-C*^Δ^*^2151-2165(NLS)^:PP:L:gfp:gpdA:nat^R^:candA-C^T^* in pUC19, *bla* | This study |
| pME4681 | Af_Δ*canA-N:ptrA-*RM in pCHS314, *bla* | This study |
| pME4682 | Vector for ectopic integration of fusion An_*candA-C1:candA-C*  *^P^candA-C1: candA-C1*^Δstop^*:candA-C:PP:L:gfp:candA-C^T^:phleo^R^* | This study |
| pME4683 | Af_*canA*^Δ838-4078^*:ptrA-*RM in pCHS314, *bla* | This study |
| pME4684 | preBiFCII cloning vector: vector:  *^T^NiiA:yfp^N^:^P^niiA/niaD:NiiD^T^:phleo^R^* in pME3741, *bla* | This study |
| pME4685 | preBiFCIII cloning vector: vector: *^T^NiiA:yfp^N^:*An_*candA‑C1^P^:niiA/niaD:NiiD^T^:phleo^R^* in pME3741, *bla* | This study |
| pME4696 | Cloning plasmid with self-excising *β-rec/six* *nat-*RM | C. Meister |
| pME4719 | BiFC vector  *^T^NiiA:yfp^N^:*An_*candA‑C1^P^niiA/niaD:yfp^C^:NiiD^T^:phleo^R^* in pME3741, *bla* | This study |
| pME4722 | *^P^candA-N:An_candA-N:candA-C:PP:L:gfp:gpdA:nat^R^:candA-C^T^* in pUC19, *bla* used as template for PCR amplification | This study |
| pME4801 | Complementation *Af_canA-N* with *mcherry:canA-N:ptrA-*RM in pCHS314, *bla* | This study |
| pME4802 | An_Cand fusion cassette:  5' flanking *candA-C1:candA-N:candA-C1:candA-C:ha*-*nat*-RM:3'flanking *candA-C* in pCHS314, *bla* | This study |
| pSK485 | self-excising *β-rec/six* *ptrA-*RM containing vector | (5) |
| pUC19L | cloning vector, linearized with *Pst*I and *Kpn*I | INVITROGEN |
