# Supplementary material for: Integration of Fungus-Specific CandA-C1 into a Trimeric CandA Complex Allowed Splitting of the Gene for the Conserved Receptor Exchange Factor of CullinA E3 Ubiquitin Ligases in Aspergilli
Source: mBio. 2019 Jun 18;10(3):e01094-19. doi: 10.1128/mBio.01094-19 (PMC6581859; doi:10.1128/mBio.01094-19)
Supplement: TABLE S5 [file mBio.01094-19-st005.docx]

**TABLE S5** *A. fumigatus* and *A. nidulans* strains used in this study. ^P^: promoter, ^T^: terminator, PP: PreScission cleavage site, L: linker, *nat*^R^: non-recyclable *nat* resistance, *phleo*^R^: non-recyclable *phleo* resistance.

| Strain | Genotype | Reference |
| --- | --- | --- |
| *Aspergillus fumigatus* strains | | |
| AfS35 (FGSC#1159) | Δ*akuA::loxP* wild type | (11) |
| AfGB76 | Δ*akuA*::*loxP*; *^P^gpdA*:*gfp*:*his2a^T^*, *ptrA^R^* | (12) |
| AfGB140 | Δ*akuA::loxP, canA*^Δ^*^exon1 (1-596):^:six* | This study |
| AfGB141 | Δ*akuA::loxP,* Δ*canA::six* | This study |
| AfGB142 | Δ*akuA::loxP, canA*^Δ^*^exon1(1-596)^::An_candA-C1:canA^exon2-4 (597-4078)^:six* | This study |
| AfGB143 | Δ*akuA::loxP, canA:: ^P^canA:canA:PP:L:gfp:six:ca*nA^T^ | This study |
| AfGB144 | Δ*akuA::loxP,* Δ*canA-N::six* | This study |
| AfGB145 | Δ*akuA::loxP,* Δ*canA^838-4078^::six* | This study |
| AfGB146 | Δ*akuA::loxP,* Δ*canA-N::six,* Δ*canA^838-4078^::six* | This study |
| AfGB172 | Δ*akuA::loxP,* Δ*canA-N::mcherry:canA-N:six* | This study |
| *Aspergillus nidulans* strains | | |
| AGB552 | ***∆****nkuA::argB, pabaA1, yA2, veA^+^* | (8) |
| AGB596 | *^P^gpdA:sgfp:phleo^R^; pabaA1*, *yA2*, *veA*^+^ | (8) |
| AGB989 | ***∆****nkuA::argB, pabaA1, yA2, veA^+^, ^P^gpdA:mrfp:h2A:hisB^T^, phleo^R^* | C. Gross, p.c. |
| AGB1014 | ***∆****nkuA::argB, pyrG89, pyroA4, veA^+^;^P^gpdA:mrfp:h2A:hisB^T,^ nat^R^* | (10) |
| AGB1094 | ***∆****nkuA::argB, pabaA1, yA2, veA^+^,* ***∆****candA-N::six* | This study |
| AGB1095 | ***∆****nkuA::argB, pabaA1, yA2, veA^+^,* ***∆****candA-N::^P^candA-N:candA-N:six:candA-N^T^* | This study |
| AGB1096 | ***∆****nkuA::argB, pabaA1, yA2, veA^+^,* ***∆****candA-C::six* | This study |
| AGB1097 | ***∆****nkuA::argB, pabaA1, yA2, veA^+^,* ***∆****candA-C::^P^candA-C:candA-C:six:candA-C^T^* | This study |
| AGB1098 | ***∆****nkuA::argB, pabaA1, yA2, veA^+^,* ***∆****candA-N::six,* ***∆****candA-C::six* | This study |
| AGB1099 | ***∆****nkuA::argB, pabaA1, yA2, veA^+^,* ***∆****candA-C1::six* | This study |
| AGB1100 | ***∆****nkuA::argB, pabaA1, yA2, veA^+^,* ***∆****candA-C1::^P^candA-C1:candA-C1:*  *PP:L:gfp:six:candA-C1^T^* | This study |
| AGB1101 | ***∆****nkuA::argB, pabaA1, yA2, veA^+^, candA-C1::5’fl candA-C1:^P^niiA/niaD::candA-C1:PP:L:gfp:six: candA-C1^T^* | This study |
| AGB1102 | ***∆****nkuA::argB, pabaA1, yA2, veA^+^, candA-C1::5’fl candA-C1:^P^niiA/niaD:candA-C1:PP:L:gfp:six: candA-C1^T^, ^P^gpdA:mrfp:h2A:hisB^T^, phleo^R^* | This study |
| AGB1103 | ***∆****nkuA::argB, pabaA1, yA2, veA^+^, ^P^gpdA:mrfp:h2A:hisB^T^, phleo^R^,*  *candA-N::^P^candA-N:gfp:PP:L:candA-N: ^P^gpdA:nat^R^:candA-N^T^* | This study |
| AGB1104 | ***∆****nkuA::argB, pabaA1, yA2, veA^+^, ^P^gpdA:mrfp:h2A:hisB^T^, phleo^R^,*  *candA-C::^P^candA-C:candA-C:PP:L:gfp:^P^gpdA:nat^R^: candA-C^T^* | This study |
| AGB1105 | ***∆****nkuA::argB, pabaA1, yA2, veA^+^,* ***∆****iORF::six* | This study |
| AGB1106 | ***∆****nkuA::argB, pabaA1, yA2, veA^+^,* ***∆****candA-C1/iORF::six* | This study |
| AGB1108 | ***∆****nkuA::argB, pabaA1, yA2, veA^+^,* ***^∆^****^ATG^candA-C1::six* | This study |
| AGB1109 | ***∆****nkuA::argB, pabaA1, yA2, veA^+^,* ***∆****candA-C1:: ^P^candA-C1::Af_canA exon1:six:candA-C1^T^* | This study |
| AGB1110 | ∆*nkuA::argB, pabaA1, yA2*, *veA*^+^, *candA-C1:iORF:candA-C::^P^candA-C1:candA-C1*^Δ^*^stop^:iORF:* ^Δ^*^start^candA-C:PP:L:gfp:six:candA-C^T^* | This study |
| AGB1111 | ***∆****nkuA::argB, pabaA1, yA2, veA^+^, ∆csnE::six* | This study |
| AGB1112 | ***∆****nkuA::argB, pabaA1, yA2, veA^+^,* ***∆****candA-N::six, ∆csnE::six* | This study |
| AGB1113 | ***∆****nkuA::argB, pabaA1, yA2, veA^+^,* ***∆****candA-C::six, ∆csnE::six* | This study |
| AGB1114 | ***∆****nkuA::argB, pabaA1, yA2, veA^+^, ∆candA-N::six,* ***∆****candA-C::six, ∆csnE::six* | This study |
| AGB1115 | ***∆****nkuA::argB, pyrG89, pyroA4, veA^+^; ^P^gpdA:mrfp:h2A:hisB^T^, nat^R^, ^P^niiA:^N^yfp:niiA^T^:^P^niiD:^C^yfp:candA-N:niiD^T^, phleo^R^* | This study |
| AGB1116 | ***∆****nkuA::argB, pyrG89, pyroA4, veA^+^; ^P^gpdA:mrfp:h2A:hisB^T^, nat^R^, ^P^niiA:^N^yfp:niiA^T^:^P^niiD:candA-C:^C^yfp:niiD^T^, phleo^R^* | This study |
| AGB1117 | ***∆****nkuA::argB, pyrG89, pyroA4, veA^+^; ^P^gpdA:mrfp:h2A:hisB^T^, nat^R^,*  *^P^niiA:candA-C1:^N^yfp:niiA^T^:^P^niiD:candA-C:^C^yfp:niiD^T^, phleo^R^* | This study |
| AGB1118 | ***∆****nkuA::argB, pyrG89, pyroA4, veA^+^; ^P^gpdA:mrfp:h2A:hisB^T^, nat^R^,*  *^P^niiA:candA-C1:^N^yfp:niiA^T^:^P^niiD:^C^yfp:candA-N:niiD^T^, phleo^R^* | This study |
| AGB1119 | ***∆****nkuA::argB, pyrG89, pyroA4, veA^+^; ^P^gpdA:mrfp:h2A:hisB^T^, nat^R^,*  *^P^niiA:candA-C1:^N^yfp:niiA^T^:^P^niiD:^C^yfp:niiD^T^, phleo^R^* | This study |
| AGB1120 | *∆nkuA::argB, pabaA1, yA2, veA^+^, ∆csnE::six,*  *^P^niiA:candA-C1:^N^yfp:niiA^T^:^P^niiD:candA-C:^C^yfp:niiD^T^, phleo^R^* | This study |
| AGB1121 | *∆nkuA::argB, pabaA1, yA2, veA^+^, ∆csnE::six,*  *^P^niiA:candA-C1:^N^yfp:niiA^T^:^P^niiD:^C^yfp:candA-N:niiD^T^, phleo^R^* | This study |
| AGB1122 | ***∆****nkuA::argB, pabaA1, yA2, veA^+^, ^P^gpdA:mrfp:h2A:hisB^T^, phleo^R^,*  *^P^candA-C:candA-C:PP:L:gfp:^P^gpdA:nat^R^: candA-C^T^, ∆candA-C1::six* | This study |
| AGB1123 | ***∆****nkuA::argB, pabaA1, yA2, veA^+^, ^P^gpdA:mrfp:h2A:hisB^T^, phleo^R^,*  *^P^candA-N:gfp:PP:L:candA-N:^P^gpdA:nat^R^:candA-N^T^, ∆candA‑C1::six* | This study |
| AGB1124 | ***∆****nkuA::argB, pabaA1, yA2, veA^+^,* ***∆****candA-C::six,*  *^P^candA-C:candA-C^∆NLS^:PP:L:gfp:^P^gpdA:nat^R^:candA-C^T^, ^P^gpdA:mrfp:h2A:hisB^T^;phleo^R^* | This study |
| AGB1125 | ***∆****nkuA::argB, pabaA1, yA2, veA^+^, ^P^gpdA:mrfp:h2A:hisB^T^, phleo^R^,*  *^P^candA-N:gfp:PP:L:candA-N: ^P^gpdA:nat^R^:candA-N^T^,*  *^P^candA‑C:candA-C^∆NLS^:six:candA-C^T^* | This study |
| AGB1127 | ***∆****nkuA::argB, pabaA1, yA2, veA^+^, 5’fl candA-C1:^P^niiA/niaD:candA-C1:PP:L:gfp:six: candA-C1^T^, ^P^gpdA:mrfp:h2A:hisB^T^ ,phleo^R^, candA-C^∆NLS^::six* | This study |
| AGB1128 | ***∆****nkuA::argB, pabaA1, yA2, veA^+^,* ***∆****candA-C::six,*  *5’fl candA‑C1:^P^niiA/niaD:candA-C1:PP:L:gfp:six:candA-C1^T^* | This study |
| AGB1129 | ***∆****nkuA::argB, pabaA1, yA2, veA^+^,* ***∆****candA-N::six,*  *5’fl candA‑C1:^P^niiA/niaD:candA-C1:PP:L:gfp:six:candA-C1^T^* | This study |
| AGB1130 | ***∆****nkuA::argB, pabaA1, yA2, veA^+^,* ***∆****candA-C::six, ,∆candA-N::six,*  *5’fl candA‑C1:^P^niiA/niaD:candA-C1:PP:L:gfp:six:candA-C1^T^* | This study |
| AGB1187 | ***∆****nkuA::argB, pabaA1, yA2, veA^+^,* ***∆****candA-C::six,*  *^P^candA-C1:candA-C1^∆stop^:∆iORF:candA-C1:PP:L:gfp:^P^gpdA:phleo^R^:trpC^T^* (ectopic) | This study |
| AGB1220 | ∆*nkuA::argB, pabaA1, yA2, veA+,* ∆*candA-N::six*, ∆c*andA-C1:candA-C::candA-N:candA-C1:candA-C:ha:six* | This study |
